# High-efficiency and stable short-delayed fluorescence emitters with hybrid long- and short-range charge-transfer excitations

**DOI:** 10.1038/s41467-023-38086-4

**Published:** 2023-04-26

**Authors:** Guoyun Meng, Hengyi Dai, Qi Wang, Jianping Zhou, Tianjiao Fan, Xuan Zeng, Xiang Wang, Yuewei Zhang, Dezhi Yang, Dongge Ma, Dongdong Zhang, Lian Duan

**Affiliations:** 1 Key Laboratory of Organic Optoelectronics, Department of Chemistry, Tsinghua University, Beijing, 100084 P. R. China; 2 Laboratory of Flexible Electronics Technology, Tsinghua University, Beijing, 100084 P. R. China; 3 Institute of Polymer Optoelectronic Materials and Devices State Key Laboratory of Luminescent Materials and Devices, South China University of Technology, Guangzhou, 510640 P. R. China

**Keywords:** Photonic devices, Organic LEDs

## Abstract

The pursuit of ideal short-delayed thermally activated delayed fluorescence (TADF) emitters is hampered by the mutual exclusion of a small singlet-triplet energy gap (Δ*E*_ST_) and a large oscillator strength (*f*). Here, by attaching an multiresonance-acceptor onto a sterically-uncrowded donor, we report TADF emitters bearing hybrid electronic excitations with a main donor-to-acceptor long-range (LR) and an auxiliary bridge-phenyl short-range (SR) charge-transfer characters, balancing a small Δ*E*_ST_ and a large *f*. Moreover, the incorporation of dual equivalent multiresonance-acceptors is found to double the *f* value without affecting the Δ*E*_ST_. A large radiative decay rate over an order of magnitude higher than the intersystem crossing (ISC) rate, and a decent reverse ISC rate of >10^6^ s^−1^ are simultaneously obtained in one emitter, leading to a short delayed-lifetime of ~0.88 μs. The corresponding organic light-emitting diode exhibits a record-high maximum external quantum efficiency of 40.4% with alleviated efficiency roll-off and extended lifetime.

## Abstract (web summary)

To possess a small singlet-triplet energy gap and a large oscillator strength simultaneously is critical for efficient thermally activated delayed fluorescent emitters. Here, the authors attach an multiresonance-acceptor onto a sterically-uncrowded donor for realizing a device efficiency of 40.4%.

## Introduction

The ability to harvest the otherwise dark triplet excitons for emission is essential to improve the efficiency of organic light-emitting diodes (OLEDs) under electrical excitation^1–3^. Following the success of organometallic complexes containing precious metals, emitters with thermally activated delayed fluorescence (TADF) have come to the forefront in recent years as their notable merits of 100% exciton utilization efficiency using noble-metal-free organic molecules^4–8^. The distinct character of a TADF emitter is its energetically close lowest-energy singlet (S_1_) and triplet (T_1_) excited states, enabling the up-conversion of dark T_1_ excitons into radiative S_1_ excitons via reverse intersystem crossing (RISC) at room temperature^9^. For an ideal TADF emitter, a fast exciton decay process is essential, which is governed by fast triplet up-conversion and singlet radiation processes simultaneously. The former requires a small singlet-triplet energy gap (Δ*E*_ST_) while the latter a large oscillator strength (*f*), which, however, are conflicting factors as they showed contrary dependence on the orbital overlap integral^10^. For most TADF emitters, long-delayed lifetimes (*τ*_D_s) of ~μs-ms are thereof observed, leading to severe bimolecular exciton annihilations under high current density^11^. Conceptually advancing molecular design strategy to break the trade-off between a small Δ*E*_ST_ and a large *f* therefore remains ongoing pursuits in literature.

To date, the traditional design principle for most TADF emitters can be simplified as donor (D)-acceptor (A) or D-π-A architectures with one or multiple donors or acceptors, of which the TADF behaviors are highly affected by the molecule electronic excitation characters. For TADF molecules with completely separated frontier molecular orbital distributions, a solely donor-to-acceptor long-range charge-transfer (LR-CT) excitation can be expected, which favors a small Δ*E*_ST_ but also a small *f*. Conventional wisdom to solve this issue is to introduce some orbital overlap on the π-bridge to hybridize localized excitation (LE) to enlarge *f* value in addition to the main LR-CT character. Nevertheless, discreetly balancing a proper portion of LR-CT and LE excitations to make a balance of a small Δ*E*_ST_ and a large *f* is rather difficult in molecular design and most emitters still face the inevitably enlarged Δ*E*_ST_s with only few emitters can reach a compromise^12,13^. Besides, an even tougher but often ignored situation is the counter-effect from the intersystem crossing (ISC) process, which can occur much more rapidly than radiative decay and thus would increase the singlet-to-triplet spin-flip transition cycles to prolong the *τ*_D_s of TADF emitters^14–16^. Therefore, though some efficient TADF emitters have been reported, most molecules with solely LR-CT or hybrid LR-CT/LE excitations still suffer from long-delayed components induced significant efficiency roll-off and poor operation stability.

Recently, multiresonance (MR) molecules have also emerged as a new family of TADF emitters by reducing Δ*E*_ST_s through the unique offset of the frontier molecular orbital distribution on single atoms^17–20^. The resulting short-range (SR) CT excitation endows MR molecules with the merits of localized states with large *f* values for extremely fast radiative decay rates (*k*_r_s), even larger than their ISC rates (*k*_ISC_s). Nonetheless, only moderate Δ*E*_ST_s can be obtained by MR emitters and according to the Marcus-Levich-Jortner theory, the small Huang-Rhys factors of those molecules also render them rather slow RISC rates (*k*_RISC_s) in the range of 10^4^ s^−1^ and even milli-second-scale *τ*_D_^21^. Inspired by the LR-CT/LE type emitters, we here envisioned that hybridizing an auxiliary SR-CT with the main LR-CT excitation may not only avoid their disadvantages but also combine their complementary advantages to afford a new paradigm of TADF molecules simultaneously with a small Δ*E*_ST_ for a fast RISC process and a large *f* for an efficient radiative decay.

**Fig. 1:**
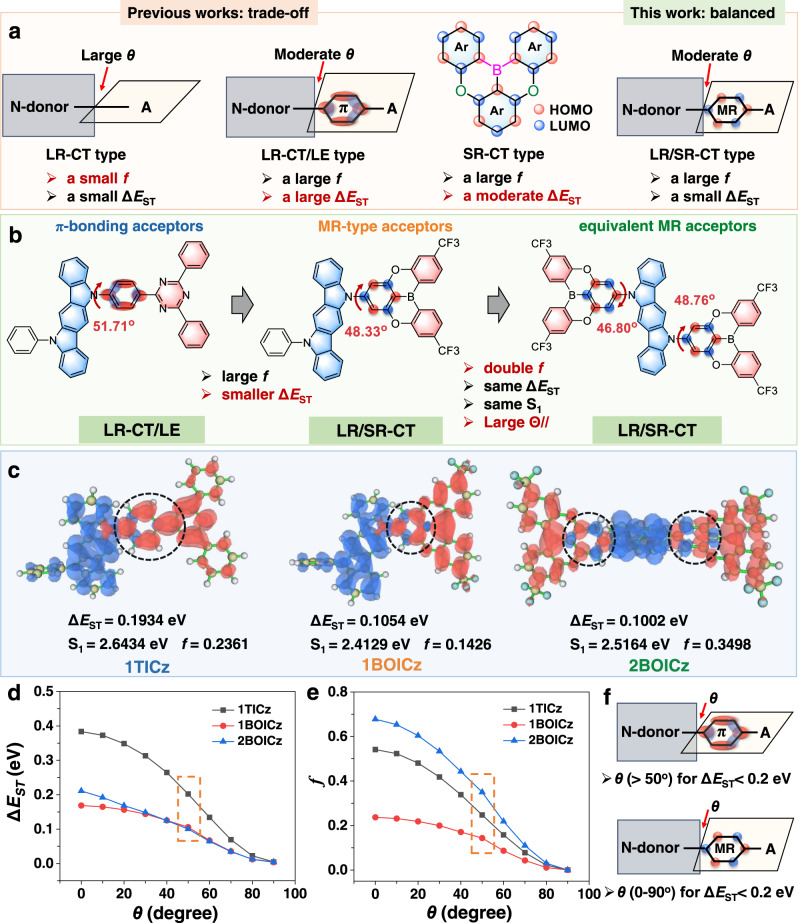
Schematic illustration of TADF molecular design strategy. **a** The design principle for TADF emitters with different electronic excitations. **b** The optimized molecular structure of 1TICz (control molecule), 1BOICz and 2BOICz. **c** The distributions of the HOMO (blue color) and LUMO (red color) and the calculated energy levels. **d**, **e** DFT-calculated values of Δ*E*_ST_ and *f* as a function of varied *θ* between donor and acceptor at the B3LYP/6-31 G(d, p) level. The dashed box corresponds to *θ* of a DFT-optimized structure for three TADF emitters in the ground state. **f** Effect of the varied *θ* between donor and acceptor on Δ*E*_ST_ for emitters with conventional acceptor and MR acceptor.

With this in mind, we demonstrated here a strategic implementation of TADF emitters with hybrid LR/SR-CT excitations by incorporating MR acceptor groups onto a sterically uncrowded donor segment (Fig. 1a). This architecture, on one hand, created a hybrid electronic excitation with a main donor-to-acceptor LR-CT and an auxiliary bridge-phenyl SR-CT characteristics, balancing a small Δ*E*_ST_ and a large *f*. On the other hand, the adoption of equivalent dual MR acceptors was unveiled to double *f* without influencing Δ*E*_ST_ and also enlarged the horizontal dipole ratio for enhanced light extraction. A large *k*_r_ of 6.0 × 10^7^ s^−1^, nearly 20 times higher than *k*_ISC_, and a decent *k*_RISC_ of 1.2 × 10^6^ s^−1^ were thereafter accomplished concurrently, establishing an ideal exciton dynamic model of *k*_r_>>*k*_ISC_ ~ *k*_RISC_ > 10^6^ s^−1^ for a short sub-microsecond-scale *τ*_D_ of 0.88 μs. The corresponding device exhibited an unprecedentedly high maximum external quantum efficiency (EQE_max_) of 40.4% with alleviated efficiency roll-off and prolonged device stability. To narrow the emission spectra, those molecules were further adopted as sensitizers for an MR emitter, achieving an EQE_max_ of 38.4% with a full-width at half-maximum (FWHM) of 25 nm. Our achievement here shatters the stereotypical physicochemical views of TADF emitters limited by the mutual exclusion of a small Δ*E*_ST_ and a large *f*, potentially revolutionizing the molecular design principle and unlocking the full potential of TADF OLEDs.

## Results and discussion

### Molecular synthesis and computational results

The structure of the proof-of-the-concept molecule, 5-(3,11-bis(trifluoromethyl)−5,9-dioxa-13b-boranaphtho[3,2,1-de]anthracene-7-yl)−11-phenyl-5,11-dihydroindolo[3,2-b]carbazole (1BOICz), was provided in Fig. 1b, constructed by attaching a trifluoromethyl (CF_3_) group substituted oxygen-bridged boron (CF_3_-BO) group on to a 5-phenyl-5,11-dihydroindolo[3,2-b]carbazole (32bICz) segment. The BO derivatives have been proven to possess obvious MR properties^17,22^ and thus were adopted as MR acceptor units. While 32bICz was a well-known donor group. For comparison, we also selected a reference TADF emitter, 5-(4-(4,6-diphenyl-1,3,5-triazin-2-yl)phenyl)−11-phenyl-5,11-dihydroindolo[3,2-b]carbazole (1TICz)^23^. We first performed the time-dependent DFT (TD-DFT) calculations to analyze the distributions of the highest occupied molecular orbital (HOMO) and the lowest unoccupied molecular orbital (LUMO) and excitation characters of those molecules (Supplementary Table 1). Derived from the sterically uncrowded structures of both donor and acceptor planes, only moderate dihedral angles (*θ*) in the range of 46-52^o^ were observed for both compounds (Fig. 1b). As a result, in addition to the mainly separated frontier molecular orbitals (FMOs) on the donor and acceptor groups, the moderate twist motifs render obvious both HOMO and LUMO residence on their phenylene bridge but in rather different behaviors (Fig. 1c). For 1TICz, a clear localized π-bonding orbital distribution was observed on the phenylene bridge with significant HOMO-LUMO overlap, thus creating a hybrid orbital distribution combining LR-CT and LE characteristics. Therefore, only a moderate Δ*E*_ST_ of 0.193 eV together with a rather high *f* value of 0.2361 was obtained for 1TICz. In terms of 1BOICz, its FMO distribution on the phenylene bridge showed a clear MR behavior with HOMO on the attached carbon atom and the carbon atoms positioned *meta* to it while the LUMO on the other carbon atoms. Such alternative electron-rich and electron-deficient regions on single atoms would thereof form the SR-CT transition for 1BOICz^22^. The natural transition orbital (NTO) analysis of S_1_ and T_1_ for both molecules was also conducted as illustrated in Supplementary Fig. 2. Clearly, in agreement with its SR-CT character on the bridge-phenyl ring, 1BOICz possessed a relatively larger ratio of CT transition than 1TICz. Interestingly, though its main CT transition, 1BOICz still exhibited a decent *f* value of 0.1428, much larger than most donor-acceptor type TADF emitters, particularly those with twisted structures^24^. The reason should be attributed to the SR-CT transition on the bridge-phenyl ring, which has proved to favor a large *f*. More intriguingly, a small Δ*E*_ST_ of 0.1054 was also observed given the small orbital overlap. Therefore, the hybrid LR/SR-CT orbital distribution characters of 1BOICz render it more advantageous than the LR-CT/LE type of 1TICz in balancing a small Δ*E*_ST_ and a large *f* value.

To compensate the sacrifice of *f* value for 1BOICz, 5,11-bis(3,11-bis(trifluoromethyl)−5,9-dioxa-13b-boranaphtho[3,2,1-de]anthracen-7-yl)−5,11-dihydroindolo[3,2-b]carbazole (2BOICz) was further developed. The FMOs of 2BOICz inherit the hybrid LR/SR-CT orbital distribution characters of 1BOICz, except for the equivalent LUMO distributions on dual acceptors. Noting that the theoretical results revealed a similar S_1_ and Δ*E*_ST_ for 2BOICz compared with that of 1BOICz, but a doubled *f* value of 0.3498 (Fig. 1c). The plausible reason should be assigned to the dual equivalent MR acceptors of 2BOICz, which can create dual equivalent emitting channels to double the *f* value. Considering that the *f* value of 2BOICz is even higher than 1TICz, this means the decreased FMO overlap by SR-CT distribution can be compensated by the equivalent acceptors without sacrificing a small Δ*E*_ST_. In our previous works, TADF emitters with multiple but not equivalent donors and/or acceptors have been developed and thus no equivalent multiple emitting channels are being formed^25^. The high *f* value of 2BOICz is even comparable with or larger than that of the conventional fluorophors with locally excited (LE) states and should lead to both fast radiative decay and RISC process combining with its small Δ*E*_ST_, which is exactly the aim of this molecular design.

To further clarify the superiority of the LR/SR-CT type orbital distributions, we simulated the corresponding Δ*E*_ST_ and *f* values by varying the *θ* between the donor and acceptor of the three compounds (Fig. 1d–f), of which the orbital distribution changes were also provided in Supplementary Fig. 3. For 1TICz, with increased *θ* value, the excitation type was changed from LR-CT/LE to solely LR-CT and thus greatly varied both Δ*E*_ST_ and *f* values. A small *θ*, that is corresponding to a hybrid LR-CT/LE excitation, would sharply increase the Δ*E*_ST_ value while a large *f*. While a large *θ*, that is corresponding to a solely LR-CT excitation, would greatly decrease the *f* value while a small Δ*E*_ST_. Balancing a proper portion of LR-CT and LE excitations to compromise a small Δ*E*_ST_ and a large *f* faces a formidable challenge in molecular design. Intriguingly, in terms of 1BOICz, a small *θ* greatly enhances the *f* value but only slightly enlarges the Δ*E*_ST_ which remained at a moderate value of ~0.16 eV even at *θ* = 0. As referred from the orbital distributions, hybrid LR/SR-CT excitations were observed at a small or moderate *θ*, suggesting that this unique excitation type can well balance a small Δ*E*_ST_ and a large *f* value in a large *θ* region. But when a large *θ* is adopted, the extension of HOMO from the donor to the phenyl linkage has vanished and only LR-CT excitations can be obtained, thus greatly reducing the *f* value. Therefore, to guarantee the hybrid short- and long-range CT distribution, a sterically uncrowded donor is crucial. In fact, this explains the reason why no LR/SR-CT excitation was observed in previously reported D-A type TADF emitters with BO-acceptors. Following the conventional wisdom, large steric N-donors were usually adopted in those emitters and thus the orbital distributions were totally separated, affording only LR-CT excitations^15,26^. Therefore, only a small *f* value can be obtained, though the small Δ*E*_ST_, rendering most of those molecules significant efficiency roll-off though some showed cutting-edge maximum EQEs.

Of particular note, 2BOICz simultaneously exhibited a small Δ*E*_ST_ similar to 1BOICz and an even larger *f* value than 1TICz, successfully breaking the mutual exclusion between a small Δ*E*_ST_ and a large *f*. Those findings provide a screen that one can construct TADF emitters with equivalent multiple acceptors (or donors) to generate equivalent multiple CT channels, which can correspondingly multiply the *f* value without influencing the small Δ*E*_ST_. To the best of our knowledge, Adachi et al. unveiled that for TADF emitters with LR-CT/LE excitations, delocalizing the frontier molecular orbital distributions would benefit to balance a large *f* and a small Δ*E*_ST_. However, the proof-of-the-concept emitters they developed only exhibited tens of microsecond-scale *τ*_D_ and moderate device efficiencies with significant efficiency roll-off^16,27^. Our molecular design principle proposed here is clearly distinguished from their strategy and opens a new pathway towards ideal TADF emitters.

### Photophysical and electronic properties

The photophysical properties of both target emitters were studied in toluene with a concentration of 10^–5^ M as depicted in Supplementary Fig. 4 and Supplementary Table 2. Similar ultraviolet-visible (UV) absorption spectra were observed for both compounds, with strong, high-energy narrowband absorption peaking at 323 nm, 339 nm, 383 nm, and 403 nm, while wide, relatively weak absorption bands at 425 nm. The former should arise from the intrinsic n-π* or π-π* transition of acceptor and donor units while the latter can be assigned to the intramolecular CT transitions. The fluorescent spectra exhibited wide and structureless green emission with peaks at 527 nm and 518 nm for 1BOICz and 2BOICz, respectively. The onset of UV-absorption spectra also defined the optical energy gap (*E*_g_) of 1BOICz (2.63 eV) and 2BOICz (2.64 eV). Meanwhile, we determined the HOMO energy levels of both compounds by ultraviolet photoemission spectroscopy (UPS) in pure neat films as illustrated in Supplementary Fig. 6. HOMO levels were estimated to be −5.89 eV for 1BOICz and −6.02 eV for 2BOICz and the LUMO energy levels thereafter can be deduced from the HOMO and *E*_g_ to be −3.26 and −3.38 eV for 1BOICz and 2BOICz, respectively.

**Fig. 2:**
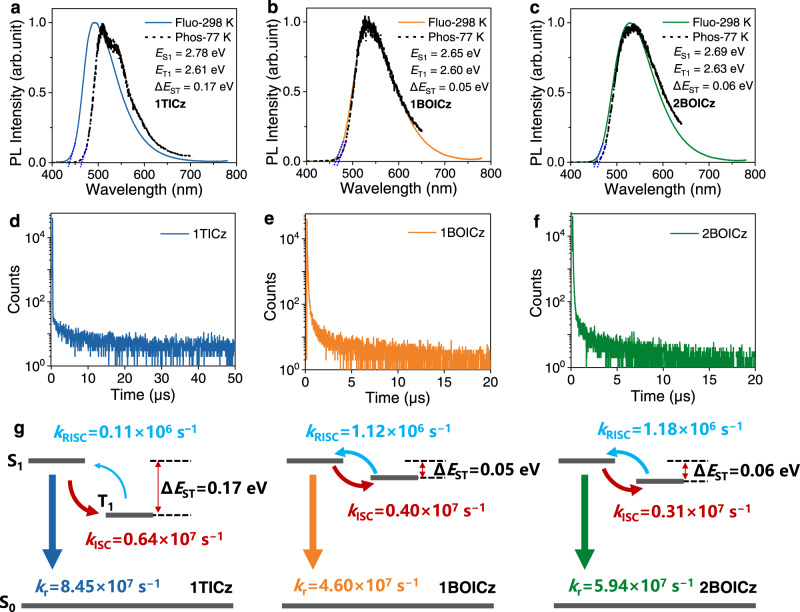
Photophysical properties of 1TICz, 1BOICz, and 2BOICz doped films. **a**–**c** Fluorescence (line, 298 K) and phosphorescence (dash dot, 77 K) spectra of their doped films (20 wt% doped films). **d**–**f** Transient PL decay curves of their doped films (20 wt% doped films). **g** Schematic illustration of decay, up-conversion, and intersystem crossing processes.

The TADF characters of both emitters were fully characterized by being dispersed in 9-(3-(9*H*-carbazol-9-yl)phenyl)−9*H*−3,9′-bicarbazole (mCPBC) matrix with a concentration of 20 wt%^28^. For comparison, the properties of 1TICz in the same conditions were also evaluated. Figure 2a–c provided the fluorescence and phosphorescence spectra of all doped films and wide structureless emissions were observed, indicating the CT characters of their S_1_ and T_1_. Therefore, the spectra onset defines the energies of S_1_ and T_1_, being 2.78 eV and 2.61 eV for 1TICz, 2.65 eV and 2.60 eV for 1BOICz and 2.71 eV and 2.65 eV for 2BOICz, respectively. Compared with the much larger Δ*E*_ST_ value of 1TICz (0.17 eV), strikingly small values of 0.05 eV for 1BOICz and 0.06 eV for 2BOICz were obtained. Those results are consistent with the theoretical results aforementioned, suggesting that the smaller Δ*E*_ST_ values of 1BOICz and 2BOICz arise from their limited HOMO-LUMO overlap by the MR-type orbital distribution on the bridge-phenyl rings.

The PL decay curves and temperature-dependent decay spectra of the three doped films were also measured under an excitation wavelength of ~365 nm as illustrated in Fig. 2d–f and Supplementary Fig. 8, all exhibiting clear TADF behaviors with both prompt and delayed components. Interestingly, unlike most TADF emitters, only rather weak delayed parts were observed and over 90% were from the prompt components. The large ratio of the prompt part evidenced that most excitons are directly radiative decay to the ground states rather than to the triplet states via ISC process. Combining with the prompt PL efficiency (*Φ*_P_) and lifetimes (*τ*_P_) of those three compounds (Table 1), large *k*_r_ values of 8.45 × 10^7^ s^−1^, 4.60 × 10^7^ s^−1^ and 5.94 × 10^7^ s^−1^ can be recorded for 1TICz, 1BOICz, and 2BOICz, respectively. The corresponding *k*_ISC_ values of 0.64 × 10^7^ s^−1^ for 1TICz, 0.40 × 10^7^ s^−1^ for 1BOICz and 0.31 × 10^7^ s^−1^ for 2BOICz were also obtained. For all compounds, the *k*_r_ is even tenfold higher than *k*_ISC_. This situation is rare for most donor-acceptor type TADF emitters, which should arise from their large *f* values. In terms of the delayed components, different from the microsecond-scale delayed lifetime of 1TICz (9.89 μs), sub-microsecond-scale delayed components were observed for 1BOICz (0.97 μs) and 2BOICz (0.88 μs), respectively. The *k*_RISC_ values of those three compounds were obtained to be 0.11 × 10^6^ s^−1^, 1.12 × 10^6^ s^−1^, and 1.18 × 10^6^ s^−1^ for 1TICz, 1BOICz, and 2BOICz, respectively. The *k*_RISC_ of 1TICz agrees with previous reports and the relatively smaller value should be due to its larger Δ*E*_ST_. Meanwhile, the emitters with MR acceptors showed nearly five times larger *k*_RISC_ values, which naturally benefit from their small Δ*E*_ST_s.

**Table 1:** Photophysical properties of 1TICz, 1BOICz, and 2BOICz in doped films

Compound	λ_PL_ (nm)^a^	*E*_S1_ (eV)^b^	*E*_T1_ (eV)^b^	Δ*E*_ST_ (eV)^b^	*Φ*_P_ (%)^c^	*Φ*_D_ (%)^c^	*τ*_P_ (ns)^d^	*τ*_D_ (ns)^d^	*k*_r_ (10^7^ s^−1^)^e^	*k*_ISC_ (10^7^ s^−1^)^e^	*k*_RISC_ (10^6^ s^−1^)^e^
1TICz	495	2.78	2.61	0.17	0.93	0.07	11	9889	8.45	0.64	0.11
1BOICz	534	2.65	2.60	0.05	0.92	0.08	20	968	4.60	0.40	1.12
2BOICz	528	2.69	2.63	0.06	0.95	0.05	16	884	5.94	0.31	1.18

^a^20 wt% doped in mCPBC matrix.

^b^Singlet (*E*_S1_) and triplet (*E*_T1_) energy levels, Δ*E*_ST_ = *E*_S1_ − *E*_T1_.

^c^Fractional quantum yields for prompt (*Φ*_P_) and delayed fluorescence (*Φ*_D_).

^d^Lifetime for prompt (*τ*_P_) and delayed (*τ*_D_) fluorescence.

^e^Rate constant of fluorescence radiative decay (*k*_r_), intersystem crossing (*k*_ISC_), and reverse intersystem crossing (*k*_RISC_), *k*_r_ = *Φ*_P_/*τ*_P_, *k*_ISC_ = (1‒*Φ*_P_)/*τ*_P_, *k*_RISC_ = *Φ*_D_/(*k*_ISC_.*τ*_P_.*τ*_D_.*Φ*_P_).

Interestingly, though their *k*_RISC_ values are not the cutting-edge ones, *τ*_D_s of 1BOICz and 2BOICz are even shorter than the ones with *k*_RISC_ > 10^7^ s^−1^^29,30^. As aforementioned, in addition to *k*_RISC_, the competition between *k*_r_ and *k*_ISC_ is also the decisive factor that controls the exciton lifetimes. The rate constants of the three compounds are illustrated in Fig. 2g. For the three compounds, the *k*_r_ values are over tenfold higher than *k*_ISC_ values and thus most S_1_ will directly decay to the ground state, rather than repeating the S_1_ ↔ T_1_ spin-flip transition cycles. Under this circumstance, the *k*_RISC_ is the true rate-determining process to control exciton lifetimes and a rate constant in the range of 10^6^ s^−1^ will lead to a sub-microsecond delayed lifetime, as is the case of 1BOICz and 2BOICz. And the even shorter *τ*_D_ of 2BOICz should arise from its larger *k*_r_, which further reduces the spin-flip transitions compared with 1BOICz. In comparison, the inefficient *k*_RISC_ of 1TICz generated a much longer delayed lifetime. Those results validate that *k*_r_>>*k*_ISC_ ~ *k*_RISC_ > 10^6^ s^−1^ is an effective dynamic model to achieve a sub-microsecond-scale delayed lifetime. Different from previous works that pursue extremely large *k*_RISC_ values, our work here provides an alternative strategy to shorten the delayed lifetime of TADF emitters. Notably that similar to 1TICz, previous works have revealed other TADF emitters possessing *k*_r_ larger than *k*_ISC_ when adopting a carbazole similar to sterically uncrowded donors^31^. However, the *k*_RISC_ values of those emitters are rather slow owing to the large Δ*E*_ST_. Taking the most representative DACT-II as an example^9^, an extremely large *k*_r_ of approaching 10^8^ s^−1^ has been obtained, which is also tenfold higher than *k*_ISC_, but a much slow *k*_RISC_ in the order of <10^5^ s^−1^. Based on the above findings, we envision that simply adopting an MR acceptor for such classic TADF emitters may unlock the full potential of their performances.

It should also be pointed out that the MR acceptor with *para*-positioned CF_3_ substituents on the B atom was first adopted among BO derivations. To illustrate the role of CF_3_, we have also constructed a reference compound (2BOICz-tBu) using 2BOICz as the model molecule while a previously reported BO-acceptor with tert-butyl groups on the *meta*-positions of B atom was adopted. The calculated electronic properties, synthesis procedure, and characterizations, and photophysical behaviors of 2BOICz-tBu were provided in the Supplementary Information. As illustrated in Supplementary Fig. 9, a similar hybrid orbital distribution was also observed for 2BOICz-tBu, suggesting that our molecular design is a universal one. But clear differences can also be observed. For 2BOICz, the orbitals on the attached phenyl rings can be well constrained on single atoms and thus a small overlap integral (0.2950) for a small Δ*E*_ST_ (0.1002). On the contrary, in terms of 2BOICz-tBu, some LUMOs on the bridge-phenyl rings would extend to the adjacent atoms where HOMOs are located, thus enlarging the orbital overlap (0.3045) for a relatively larger Δ*E*_ST_ (0.1270). This discrepancy proved that the existence of CF_3_ groups could enhance the MR-type LUMO distributions on the attached phenyl rings. Besides, owing to the weak electron-withdrawing ability of the tert-butyl units substituted BO-acceptor, 2BOICz-tBu showed a significantly blue-shifted fluorescence emission with a high S_1_ energy of 2.90 eV. Moreover, a well-resolved phosphorescence spectrum resembling that of the donor segment was recorded, suggesting its LE character of triplet state with an energy of 2.65 eV. A large Δ*E*_ST_ of 0.25 eV was thereafter obtained, affording rather poor TADF properties. Contrarily, adopting the CF_3_ substituted on acceptors of 2BOICz greatly reduced the energy of its CT singlet and CT triplet states, avoiding the influence of low-lying LE triplet and thus favoring a small Δ*E*_ST_ for efficient RISC.

Based on those findings, we found that serval criteria should be satisfied to maximize the performances of TADF emitters with LR/SR-CT excitations. Firstly, a moderate dihedral angle between the donor and MR acceptor segments is necessary to guarantee HOMO extension to the bridge-phenyl rings. Secondly, the LE triplet states of the donor and MR acceptor should be energetically close to or higher than CT states to avoid its influence on Δ*E*_ST_s^32^. Thirdly, proper substitutions on MR-acceptors should be considered to enhance the MR characters to guarantee the SR-CT excitations on the bridge-phenyl rings. It should be pointed out that some cutting-edge molecules in literature with mainly LR-CT transitions have also realized a short-delayed lifetime of <1 μs by modulating the energy levels of LE triplet (^3^LE) and CT singlet (^1^CT) excited states^29,33,34^. Our molecular design here is totally different with previous works as what we tried to manipulate is the orbital distribution type. And those previously reported strategies to enhance the RISC process should also work for our emitters, which may further improve the performances of LR/ST-CT emitters.

### Device characterization and performance

**Fig. 3:**
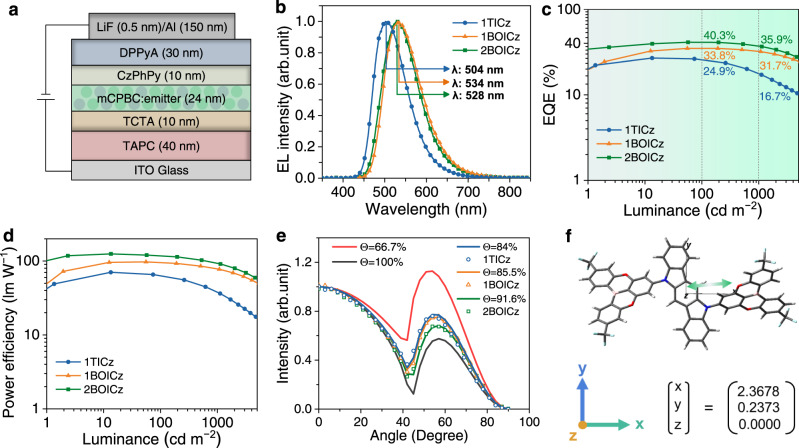
Device structure and performance of OLEDs. **a** Architectures of the devices. **b** Normalized EL spectra under 1000 cd m^−2^. **c** EQE versus luminance characteristics. **d** Power efficiency versus luminance characteristics. **e** Angle-dependent PL spectra of 2BOICz, 1BOICz and 1TICz doped in mCPBC host. **f** The direction of the calculated S_0_-S_1_ transition dipole moment (as indicated by the arrow) of 2BOICz.

The electroluminescence (EL) performances of both emitters were further evaluated using the following device architecture: Indium tin oxide (ITO)/TAPC (4,4′-cyclohexylidenebis[N,N-bis(4-methylphenyl)benzenamine]) (40 nm)/TCTA (tris(4-(9H-carbazol-9-yl)phenyl)amine) (10 nm)/mCPBC: emitters (24 nm)/CzPhPy (4,6-bis(3-(9H-carbazol-9-yl)phenyl)pyrimidine) (10 nm)/DPPyA (9,10-bis(6-phenylpyridin-3-yl)anthracene) (30 nm)/LiF (lithium fluoride) (0.5 nm)/Al (150 nm). The concentrations of those emitters were optimized to be 20 wt% (Supplementary Table 3–5) and the diagram of the device architectures is illustrated in Fig. 3a. For comparison, the control device with 1TICz was constructed. The EL spectra recorded at 1000 cd m^−2^ were provided in Fig. 3b and showed wide emission with peaks at 534 nm for 1BOICz, 528 nm for 2BOICz, and 504 nm for 1TICz, corresponding to Commission Internationale de l´Eclairage (CIE) coordinates of (0.381, 0.566), (0.383, 0.550) and (0.247, 0.499), respectively, which consisted with the PL spectra of the doped films, indicating the complete energy transfer. Supplementary Figs. 12–17 revealed an interesting phenomenon that both 1BOICz and 2BOICz-based devices exhibited obviously red-shifted emissions with increased dopant concentrations while those one with 1TICz only showed limited red-shift. This can be well explained by the much larger excited-state dipole moments of 1BOICz (1.11 D) and 2BOICz (1.16 D) than that of 1TICz (0.37 D), induced by the strong electron-withdrawing ability of CF_3_ substituted BO-acceptors.

The EQE versus luminance plots of those devices were illustrated in Fig. 3c, a significantly improved EQE_max_ up to 34.6% was obtained for 1BOICz, much higher than that of 1TICz (26.1%). A greatly alleviated efficiency roll-off was also noted for 1BOICz-based device, with EQE values of 33.8% and 31.7% at high luminance of 100 cd m^−2^ and 1000 cd m^−2^. On the contrary, the EQE of 1TICz-based device sharply decreased to 24.9% and 16.7% at 100 cd m^−2^ and 1000 cd m^−2^. The differences in efficiency roll-off behaviors were believed to be originated from the TADF emissive dynamics of the two emitters under electrical excitation, which will be depicted later by EL decay curves. Notably, 2BOICz-based device showed an exceptionally high EQE_max_ of 40.4%, calibrated using the angle-dependent EL distribution (Supplementary Fig. 18), which was maintained at 40.3% and 35.9% at 100 cd m^−2^ and 1000 cd m^−2^, respectively. A maximum power efficiency (PE_max_) of 122.4 lm W^−1^ was also observed for 2BOICz as illustrated in Fig. 3d, obviously outperforming those of 1BOICz (95.5 lm W^−1^) and 1TICz (71.7 lm W^−1^), respectively. We have also measured the angle-dependent EL intensities of the devices based on 1TICz, 1BOICz, and 2BOICz, and nearly Lambertian profiles pattern with the Lambertian coefficients of 0.97, 0.98 and 0.98 were recorded (Supplementary Fig. 18), respectively. Especially, the calibrated EQE_max_ value of 2BOICz device was still up to 40%, indicating that the high device efficiency was not overestimated. To confirm the reproducibility of the peak EQE_max_ of the device based on 2BOICz, ten groups of devices (40 testing points) were fabricated in parallel. The statistics of those EQE_max_s show that the device performance is in good reproducibility.

To better understand the origin of the impressive efficiencies of those devices, we investigated the angle-dependent *p*-polarization-resolved PL intensity measurement of the corresponding emitting layers. The varied PL intensity measured with different angles was given in Fig. 3e and was analyzed with the classical dipole optical simulations. A high ratio of horizontal emitting dipole orientation (Θ_//_) of 85.5% and 84.0% were obtained for 1BOICz and 1TICz, which was further increased to 91.6% for 2BOICz. Owing to the moderate dihedral angles between donor and acceptors, those molecules possess large quasi-planar structures, which were further enlarged by the dual equivalent acceptors. The large molecular planarity should render the molecular orientation parallel to the base plane during the evaporation process. Meanwhile, the calculated S_0_-S_1_ transition dipole moment as illustrated in Fig. 3f and Supplementary Fig. 19 is nearly parallel to the molecular orientation for all three compounds, thus generating those high Θ_//_s. Previous work has validated the critical role of a high Θ_//_ in enhancing the light outcoupling efficiency for OLEDs^35–37^. The highest Θ_//_ of 2BOICz among the three compounds also accounts for its highest efficiency. Besides, the low refractive index of the hole-transporting material, TAPC, is also beneficial to this high EQE. As a comparison, we have also fabricated a device with another most commonly used hole-transporting material, NPB, which possesses a relatively higher refractive index, and a relatively lower EQE_max_ of 38% was realized (Supplementary Fig. 20). Therefore, the excellent TADF properties, a high Θ_//_ and an optimized device structure should concurrently account for the cutting-edge EQE_max_ of 2BOICz. This is in accordance with the theoretically predicted EQE efficiency performed by commercial software OFSS 1.0^38,39^ (Wuhan Yuwei Optical Software Co., Ltd.) (Supplementary Fig. 21). In fact, previous works have also demonstrated EQE_max_s of over 40% using TADF emitters with similar *Φ* and Θ_//_ values as illustrated in Supplementary Table 7. It is interesting to note that most of those previously reported emitters showed relatively larger efficiency roll-off compared with 2BOICz, though their similar EQE_max_s. This further evidenced the advantages of our emitters with hybrid LR/SR-CT excitations to achieve a short-delayed component for an alleviated efficiency roll-off.

**Fig. 4:**
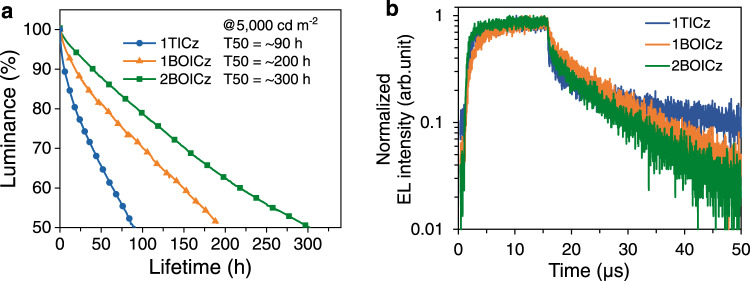
Stability and EL transient characteristics of TADF devices. **a** The operational lifetimes of the devices, and **b** EL transient spectra of devices based 1TICz, 1BOICz, and 2BOICz.

We further evaluated the operational stabilities of those devices at a constant current with an initial luminance of 5000 cd m^−2^ as shown in Fig. 4a. Decent half-lifetimes (LT50s) of 89 h, 196 h and 302 h were obtained for 1TICz, 1BOICz, and 2BOICz-based devices, respectively. Using a commonly adopted degradation acceleration factor (n) of 1.75, a LT50 at an initial luminance of 1000 cd m^−2^ can be extrapolated with the equation of LT50 (1000 cd m^−2^) = LT50 (5000 cd m^−2^) × (5000 cd m^−2^/1000 cd m^−2^)^n^, being 1486 h for 1TICz, 3272 h for 1BOICz and 5043 h for 2BOICz, respectively. Notably that the longest lifetime was observed for 2BOICz, over three times longer than that of 1TICz, evidencing that the molecular design strategy possesses the potential to improve device stability compared with the conventional ones^40^.

To understand the underlying physics of the alleviated efficiency roll-off and extended operational stability of the two novel emitters under electrical excitation, the EL decay curves of those devices under an operation voltage of 6 V were recorded. It should be further noted that different to the situation in PL excitations, 75% of the generated excitons under EL excitations would be triplet ones. Therefore, the ratio of the delayed component will greatly be enlarged and a balance in *k*_r_, *k*_ISC_, and *k*_RISC_ to short *τ*_D_ would matter more for device performances. As provided in Fig. 4b, the *τ*_D_s of all three compounds showed a trend of 1TICz > 1BOICz > 2BOICz, which is exactly inverse to their LT50s. As aforementioned, various bimolecular annihilation processes have been acknowledged as the dominant reasons for not only the efficiency roll-off under high luminance but also the operation stability^11,41^. Briefly, those bimolecular annihilation processes strongly depend on both concentrations and residence time of excitons. The small efficiency roll-off and the longer LT50s of both 1BOICz and 2BOICz should arise from their shorter delayed lifetimes than 1TICz, which can greatly suppress exciton annihilations under EL excitation.

**Fig. 5:**
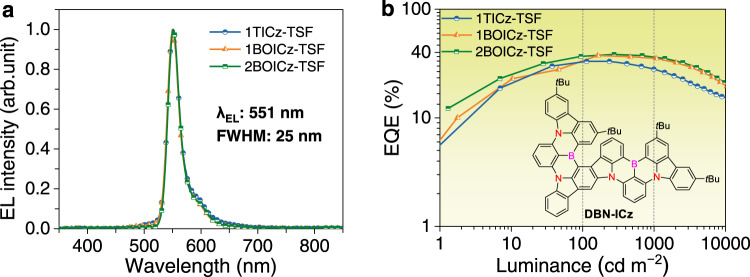
TSF-OLED device performances. **a** Normalized EL spectra under 1000 cd m^−2^. **b** EQE versus luminance characteristics, and the narrowband yellow MR emitter DBN-ICz.

Besides being directly adopted as emitters, TADF molecules have also been widely used as sensitizers for narrowband MR emitters to assist the recycling of triplet states, known as hyperfluorescence or TADF-sensitized fluorescence (TSF). As an ideal sensitizer, a fast RISC process is required to enhance exciton utilization and a quick radiative decay is also necessary to guarantee the desired Förster energy transfer (FET). The well-balanced *k*_r_ and *k*_RISC_ of the LR/SR-CT type TADF molecules, therefore, render them good sensitizers for MR emitters. To validate this inspiration, the performances of 1BOICz and 2BOICz as sensitizers were evaluated using DBN-ICz as the MR emitter^42^. For comparison, 1TICz was also adopted as a sensitizer. The detailed photophysical properties and energy transfer processes were given in Supplementary Fig. 23. All three sensitizers provided efficient spectral overlapping between their emission spectra with the absorption spectrum of DBN-ICz emitter, affording large FET radius (*R*_0_) of 3.4, 3.3, and 3.3 nm for 1TICz, 1BOICz, and 2BOICz-based systems, respectively, implying the efficient energy transfer in these sensitizing systems. TSF-OLEDs were fabricated with mCPBC: 20 wt% sensitizers: 1 wt% DBN-ICz as EMLs while all the other functional layers were similar to the TADF devices. Figure 5a provides the EL spectra of those three devices, all showing narrowband emissions with a peak at 551 nm, a small FWHM of 25 nm, and CIE coordinates of (0.37, 0.61). Clear differences were observed from their EQE-luminance characteristics as displayed in Fig. 5b. The 1BOICz and 2BOICz-based TSF devices achieved high EQE_max_ values of 36.6% and 37.6%, respectively, with greatly reduced efficiency roll-off (Table 2). On the contrary, 1TICz-based TSF device displayed not only a relatively lower EQE_max_ (32.2%) but also a larger efficiency roll-off. The EL decay curves as illustrated in Supplementary Fig. 24 and depicted relatively shorter lifetimes for devices based on 1BOICz and 2BOICz, which should benefit to suppress exciton annihilations under high luminance and thus lower efficiency roll-off than the device using 1TICz. The quick exciton consumption of 1BOICz and 2BOICz-based devices should also be assigned to the more balanced *k*_RISC_ and *k*_r_ from the LR/SR-CT orbital distributions.

**Table 2:** Summary of the EL performance of TADF-OLEDs and TSF-OLEDs devices

Device	Compound	λ_EL_^a^ [nm]	FWHM [nm]	V_on_^b^ [V]	EQE_max/100/1000_^c^ [%]	PE_max/100/1000_^d^ [lm W^−1^]	CIE (x,y)^e^
	1TICz	504	100	3.1	26.1/24.9/16.7	71.7/61.6/37.1	(0.247, 0.499)
TADF^f^	1BOICz	534	101	3.1	34.6/33.8/31.7	95.5/92.1/76.0	(0.381, 0.566)
	2BOICz	528	91	3.0	40.4/40.3/35.9	122.4/114.4/89.6	(0.383, 0.550)
	1TICz	551	25	3.0	32.2/32.1/26.3	88.0/87.4/60.9	(0.371, 0.611)
TSF^f^	1BOICz	551	25	3.0	36.6/32.9/34.4	99.3/94.5/87.5	(0.372, 0.612)
	2BOICz	551	25	3.0	37.6/36.2/35.4	102.3/101.2/89.4	(0.372, 0.613)

^a^EL peak wavelength.

^b^Turn-on voltage (V_on_).

^c^Maximum external quantum efficiency (EQE), value at 100 and 1000 cd cm^−2^.

^d^Maximum power efficiency (PE), value at 100 and 1000 cd cm^−2^.

^e^CIE coordinates at 1000 cd cm^−2^.

^f^Both PE and EQE values of TADF and TSF devices are calibrated by Lambertian correction.

## Discussion

In summary, a novel LR/SR-CT type TADF molecule design principle was proposed, breaking the trade-off between a small Δ*E*_ST_ and a large *f* towards the goal of a short *τ*_D_. By attaching equivalent MR acceptors onto a sterically uncrowded nitrogen-donor, this architecture not only creates a hybrid orbital distribution involving a hybrid donor-to-acceptor LR-CT and bridge-phenyl SR-CT characteristics to balance a large *f* and a small Δ*E*_ST_, but also allows equivalent radiative channels to further enlarge the *f* without affecting the Δ*E*_ST_. The proof-of-the-concept emitter, 2BOICz, displayed well-balanced rate constants to establish an exciton dynamic model of *k*_r_>>*k*_ISC_ ~ *k*_RISC_>>10^6^ s^−1^ for an extraordinarily fast emission lifetime of 0.88 μs. The quasi-planar structure of this emitter also favors essentially high Θ_//_ for a large outcoupling efficiency and the corresponding device exhibited a remarkably high EQE_max_ of 40.4%, which remained at 35.9% at 1000 cd m^−2^ with an extended LT50 of over 5000 h simultaneously. The balanced fast RISC and radiative decay also render this compound an ideal sensitizer for MR emitter and a high EQE_max_ of 37.6% with alleviated efficiency roll-off was realized with an extremely small FWHM of 25 nm. It is also envisioned that within the prerequisite of maintaining a larger *k*_r_ than *k*_ISC_, the *k*_RISC_ may be further improved by enlarging the spin-orbital coupling (SOC) by introducing heavy atoms and modulating the energy levels of the localized and CT states. A potential molecular design is to adopt sulfur-bridged boron (BS) MR acceptors. Considering that previous D-A type TADF emitters always bear LR-CT or LR-CT/LE type orbital distributions, we believe that the LR/SR-CT type proposed here should represent a concept advancement towards a new paradigm of TADF emitters with short-delayed components.

## Methods

The synthetic procedures of both boron-based compounds were illustrated in supporting information. The intermediate BO-Br was prepared using the previously reported cyclization reactions in the presence of n-butyllithium (*n*-BuLi) and boron tribromide (BBr_3_). Afterward, the Buchwald-Hartwig amination was conducted to synthesize the final compounds with high yields of over 70%. The final products were further purified using temperature-gradient vacuum sublimation, of which the chemical structures were then fully characterized using ^1^H nuclear magnetic resonance (NMR) spectroscopy, mass spectrometry (MS), and elemental analysis. Detailed synthetic procedures and characterization data are provided in the supplementary information.

## Supplementary information

Supplementary Information

Peer Review File

## Data Availability

The data supporting the findings of this study are available within the paper and the Supplementary Information. Source data are provided with this paper.

## Source data

Source Data

## References

[1] Tang CW, VanSlyke SA (1987). Organic electroluminescent diodes. Appl. Phys. Lett..

[2] Sato K (2013). Organic luminescent molecule with energetically equivalent singlet and triplet excited states for organic light-emitting diodes. Phys. Rev. Lett..

[3] Uoyama H, Goushi K, Shizu K, Nomura H, Adachi C (2012). Highly efficient organic light-emitting diodes from delayed fluorescence. Nature.

[4] Baldo MA (1998). Highly efficient phosphorescent emission from organic electroluminescent devices. Nature.

[5] Goushi K, Yoshida K, Sato K, Adachi C (2012). Organic light-emitting diodes employing efficient reverse intersystem crossing for triplet-to-singlet state conversion. Nat. Photonics.

[6] Lee J (2016). Deep blue phosphorescent organic light-emitting diodes with very high brightness and efficiency. Nat. Mater..

[7] Fu H, Cheng Y-M, Chou P-T, Chi Y (2011). Feeling blue? Blue phosphors for OLEDs. Mater. Today.

[8] Sun J (2022). Exceptionally stable blue phosphorescent organic light-emitting diodes. Nat. Photonics.

[9] Kaji H (2015). Purely organic electroluminescent material realizing 100% conversion from electricity to light. Nat. Commun..

[10] Zhang D (2014). High-efficiency fluorescent organic light-emitting devices using sensitizing hosts with a small singlet-triplet exchange energy. Adv. Mater..

[11] Murawski C, Leo K, Gather MC (2013). Efficiency roll-off in organic light-emitting diodes. Adv. Mater..

[12] Chen XK (2017). A new design strategy for efficient thermally activated delayed fluorescence organic emitters: from twisted to planar structures. Adv. Mater..

[13] Liu YC, Li CS, Ren ZJ, Yan SK, Bryce MR (2018). All-organic thermally activated delayed fluorescence materials for organic light-emitting diodes. Nat. Rev. Mater..

[14] Yin C (2022). Highly efficient and nearly roll-off-free electrofluorescent devices via multiple sensitizations. Sci. Adv..

[15] Kim JU (2020). Nanosecond-time-scale delayed fluorescence molecule for deep-blue OLEDs with small efficiency rolloff. Nat. Commun..

[16] Hirata S (2015). Highly efficient blue electroluminescence based on thermally activated delayed fluorescence. Nat. Mater..

[17] Hirai H (2015). One-step borylation of 1,3-diaryloxybenzenes towards efficient materials for organic light-emitting diodes. Angew. Chem. Int. Ed..

[18] Hatakeyama T (2016). Ultrapure blue thermally activated delayed fluorescence molecules: efficient HOMO-LUMO separation by the multiple resonance effect. Adv. Mater..

[19] Kondo Y (2019). Narrowband deep-blue organic light-emitting diode featuring an organoboron-based emitter. Nat. Photonics.

[20] Pershin A (2019). Highly emissive excitons with reduced exchange energy in thermally activated delayed fluorescent molecules. Nat. Commun..

[21] Liu J (2022). Toward a BT.2020 green emitter through a combined multiple resonance effect and multi-lock strategy. Nat. Commun..

[22] Ikeda N (2020). Solution-processable pure green thermally activated delayed fluorescence emitter based on the multiple resonance effect. Adv. Mater..

[23] Xiang S (2018). To improve the efficiency of thermally activated delayed fluorescence OLEDs by controlling the horizontal orientation through optimizing stereoscopic and linear structures of indolocarbazole isomers. J. Mater. Chem. C..

[24] Tao Y (2014). Thermally activated delayed fluorescence materials towards the breakthrough of organoelectronics. Adv. Mater..

[25] Zhang D (2020). Efficient and stable deep-blue fluorescent organic light-emitting diodes employing a sensitizer with fast triplet upconversion. Adv. Mater..

[26] Ahn DH (2019). Highly efficient blue thermally activated delayed fluorescence emitters based on symmetrical and rigid oxygen-bridged boron acceptors. Nat. Photonics.

[27] Tanaka M, Nagata R, Nakanotani H, Adachi C (2020). Understanding degradation of organic light-emitting diodes from magnetic field effects. Commun. Mater..

[28] Wei P, Zhang D, Duan L (2019). Modulation of Förster and Dexter interactions in single‐emissive‐layer all‐fluorescent WOLEDs for improved efficiency and extended lifetime. Adv. Funct. Mater..

[29] Wada Y, Nakagawa H, Matsumoto S, Wakisaka Y, Kaji H (2020). Organic light emitters exhibiting very fast reverse intersystem crossing. Nat. Photonics.

[30] Matsuo K, Yasuda T (2019). Blue thermally activated delayed fluorescence emitters incorporating acridan analogues with heavy group 14 elements for high-efficiency doped and non-doped OLEDs. Chem. Sci..

[31] Kim HJ (2021). Ultra‐deep‐blue aggregation‐induced delayed fluorescence emitters: achieving nearly 16% EQE in solution‐processed nondoped and doped OLEDs with CIEy<0.1. Adv. Funct. Mater..

[32] Cheon HJ, Woo SJ, Baek SH, Lee JH, Kim YH (2022). Dense local triplet states and steric shielding of a multi-resonance TADF emitter enable high-performance deep-blue OLEDs. Adv. Mater..

[33] Lee YH (2021). Managing local triplet excited states of boron-based TADF emitters for fast spin-flip process: Toward highly efficient TADF-OLEDs with low efficiency roll-off. Chem. Eng. J..

[34] Aizawa N, Matsumoto A, Yasuda T (2021). Thermal equilibration between singlet and triplet excited states in organic fluorophore for submicrosecond delayed fluorescence. Sci. Adv..

[35] Chen Y (2021). Approaching nearly 40% external quantum efficiency in organic light emitting diodes utilizing a green thermally activated delayed fluorescence emitter with an extended linear donor-acceptor-donor structure. Adv. Mater..

[36] Lee Y, Hong JI (2021). High‐efficiency thermally activated delayed fluorescence emitters with high horizontal orientation and narrow deep‐blue emission. Adv. Opt. Mater..

[37] Lim H (2020). Highly efficient deep-blue OLEDs using a TADF emitter with a narrow emission spectrum and high horizontal emitting dipole ratio. Adv. Mater..

[38] Chen L (2022). Multi-parameter and multi-objective optimization of stratified OLEDs over wide field-of-view considering thickness tolerance. Opt. Express.

[39] Ke X (2019). Simulation method for study on outcoupling characteristics of stratified anisotropic OLEDs. Opt. Express.

[40] Kim M, Jeon SK, Hwang SH, Lee JY (2015). Stable blue thermally activated delayed fluorescent organic light-emitting diodes with three times longer lifetime than phosphorescent organic light-emitting diodes. Adv. Mater..

[41] Sudheendran Swayamprabha S (2020). Approaches for long lifetime organic light emitting diodes. Adv. Sci..

[42] Zhang, Y. et al. Multiple fusion strategy for high-performance yellow OLEDs with full width at half maximums down to 23 nm and external quantum efficiencies up to 37.4. *Adv. Mater*. **35**, e2209396 (2022). 10.1002/adma.202209396 36435993

