# Supplementary material for: High-efficiency and stable short-delayed fluorescence emitters with hybrid long- and short-range charge-transfer excitations
Source: Nat Commun. 2023 Apr 26;14:2394. doi: 10.1038/s41467-023-38086-4 (PMC10133342; doi:10.1038/s41467-023-38086-4)
Supplement: Supplementary file 1 — Supplementary Information [file 41467_2023_38086_MOESM1_ESM.pdf]

Supplementary Information

High-efficiency and stable short-delayed fluorescence emitters with hybrid long- and short-range charge-transfer excitations

Guoyun Meng,[1] *Hengyi Dai,*[1] *Qi Wang,*[1] *Jianping Zhou,*[1] *Tianjiao Fan,*[1] *Xuan Zeng,*[1] *Xiang Wang,*[1] *Yuewei Zhang,*[1,2] *Dezhi Yang,*[3] *Dongge Ma,*[3] *Dongdong Zhang,*[*1,2] *and Lian Duan*[*1,2]

[1] Key Laboratory of Organic Optoelectronics, Department of Chemistry, Tsinghua University, Beijing 100084, P. R. China

[2] Laboratory of Flexible Electronics Technology, Tsinghua University, Beijing 100084, P. R. China

[3] Institute of Polymer Optoelectronic Materials and Devices State Key Laboratory of Luminescent Materials and Devices, South China University of Technology, Guangzhou, 510640, P. R. China

*E-mail: ddzhang@mail.tsinghua.edu.cn; duanl@mail.tsinghua.edu.cn

Table of Contents

1. Supplementary Figures **3**

2. Supplementary Tables **23**

3. Supplemental Methods **26**

3.1 General information 26

3.2 Computational methods 26

3.3 Measurement of absorption and emission characteristics 27

3.4 Ultraviolet photoelectron spectroscopy (UPS) characterizations 27

3.5 The rate constants calculation 27

3.6 Determination of the emitting dipole orientation 27

3.7 Device fabrication and measurement of EL characteristics ..28

3.8 Synthesis 28

4. Supplementary References **31**

1. Supplementary Figures

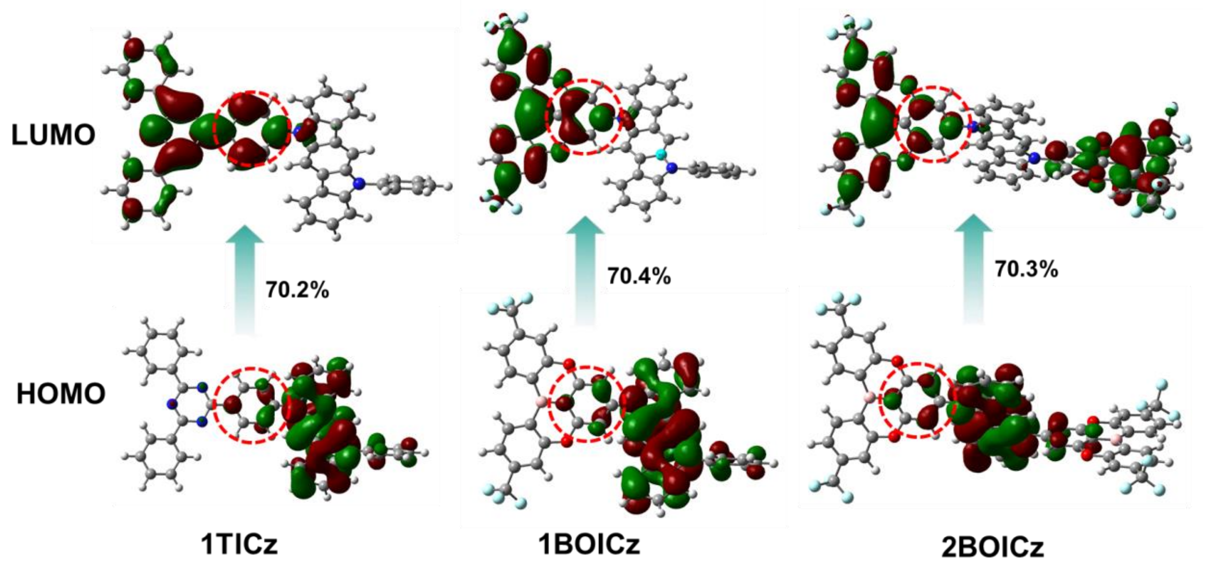

Supplementary Fig. 1 Distributions of HOMO and LUMO of 1TICz, 1BOICz and 2BOICz.

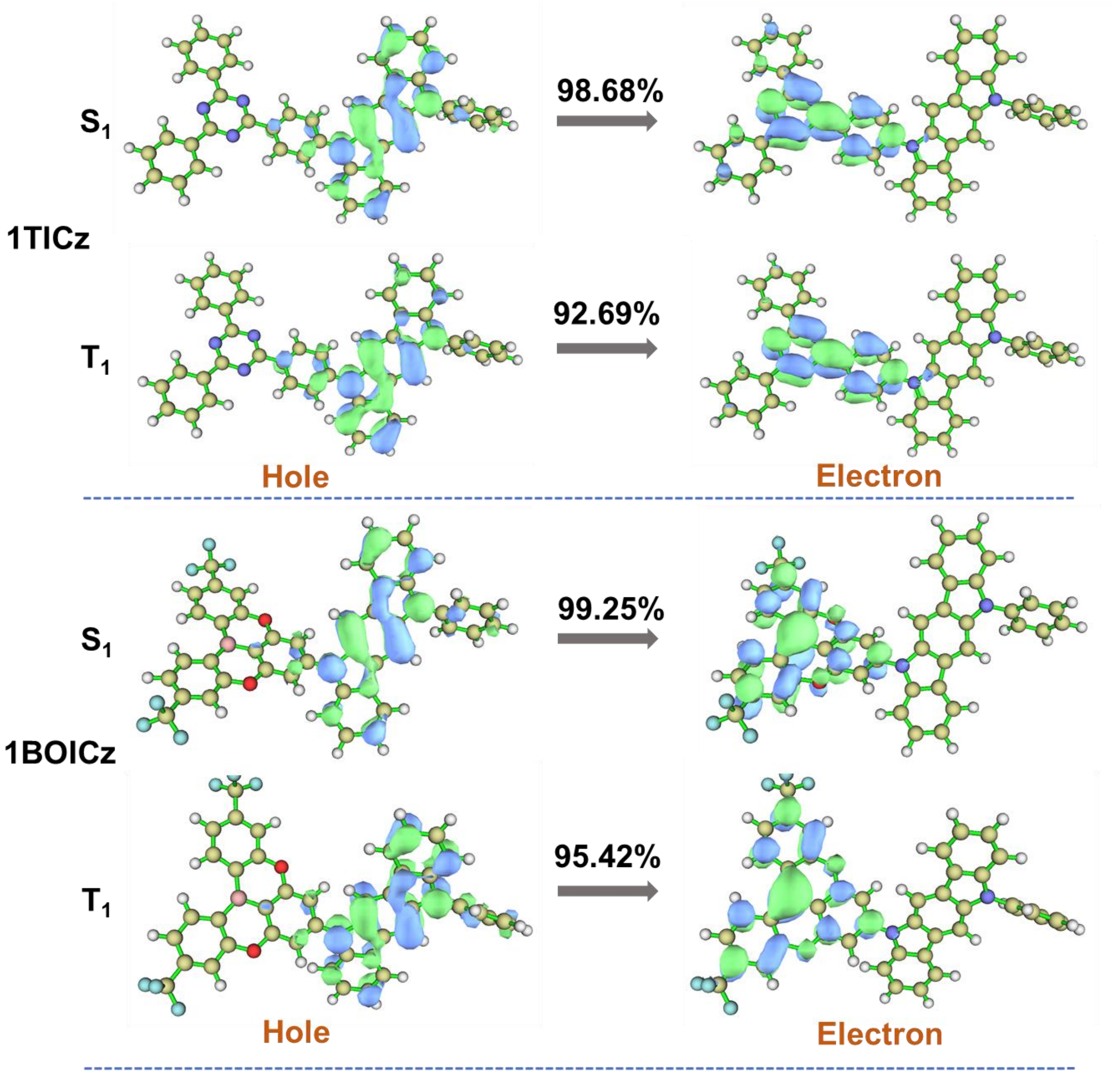

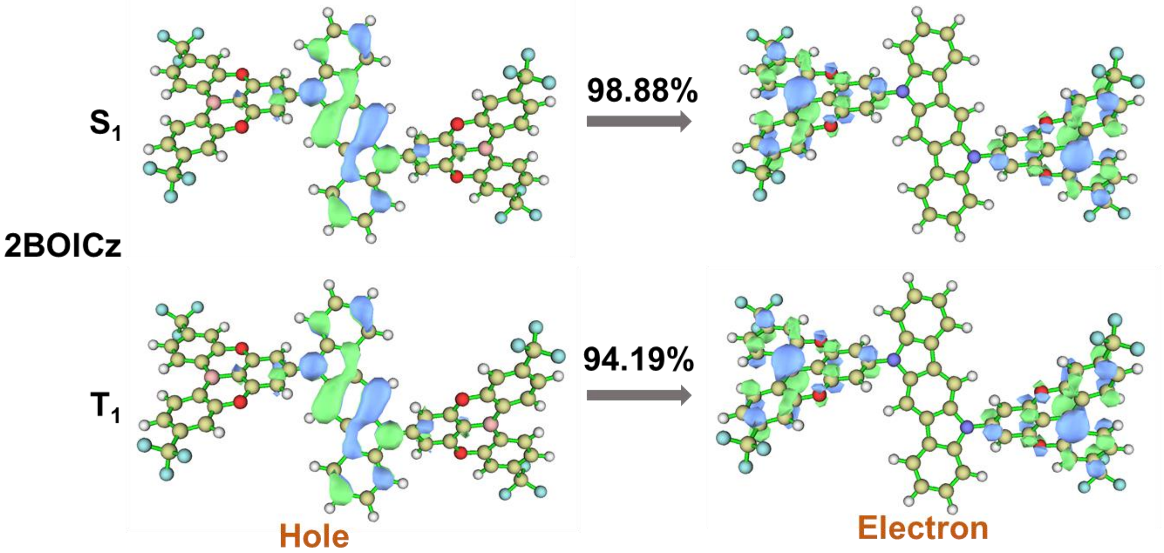

Supplementary Fig. 2 Natural transition orbitals (NTOs) describing the excitation characters of the S_1, T_1 and T_2 state in 1TICz, 1BOICz and 2BOICz; the weights of the hole-electron contributions to the excitations are included. For these three compounds, the transition of both S_1 and T_1 states is dominated by hole→electron (> 92%) with CT characters.

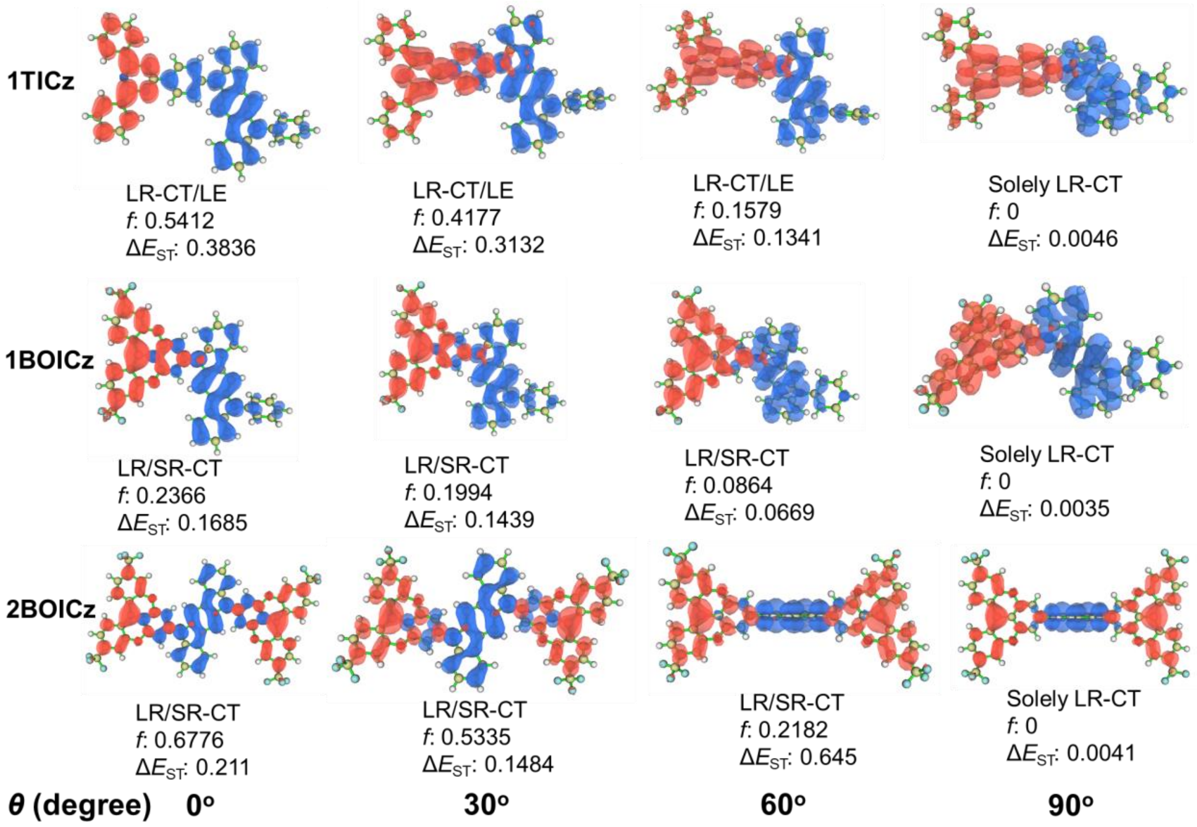

Supplementary Fig. 3 Effect of the varied θ between donor and acceptor on HOMOs (blue color) and LUMOs (red color) for 1TICz, 1BOICz and 2BOICz.

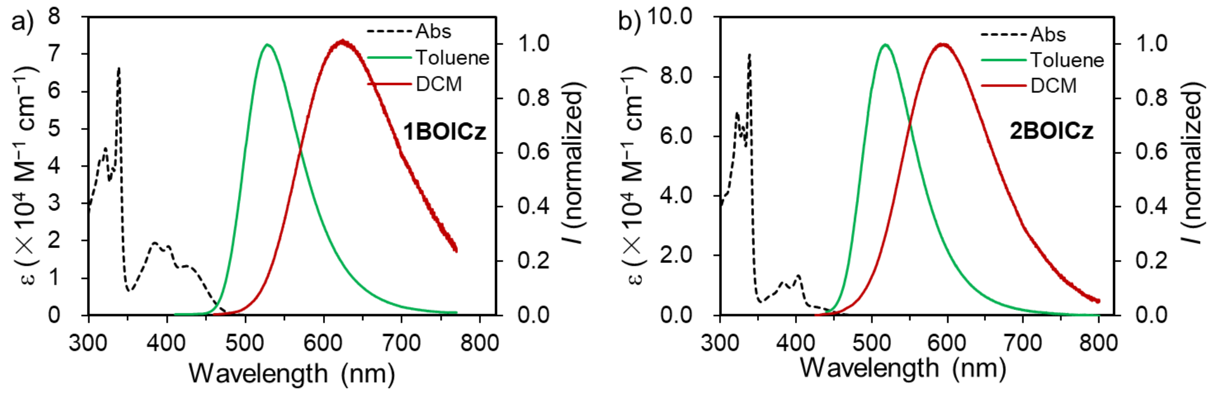

Supplementary Fig. 4 Ultraviolet-visible absorption (left axis) in toluene (10^{-5} M), and normalized PL spectra (right axis) of (a) 1BOICz and (b) 2BOICz in toluene and DCM (10^{-5} M), respectively.

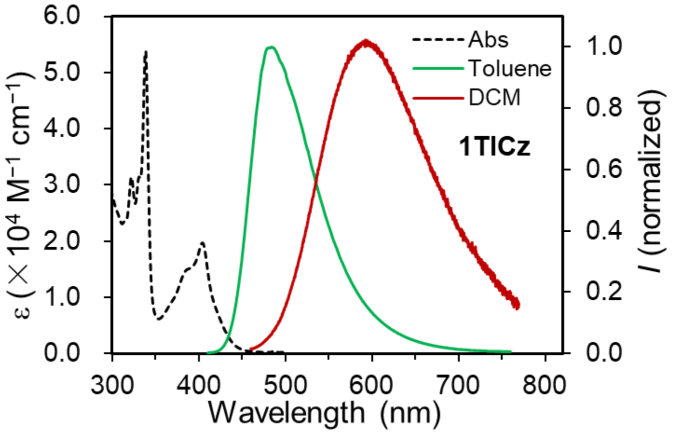

Supplementary Fig. 5 Ultraviolet-visible absorption (left axis) in toluene (10^{-5} M), and normalized PL spectra (right axis) of 1TICz in toluene and DCM (10^{-5} M).

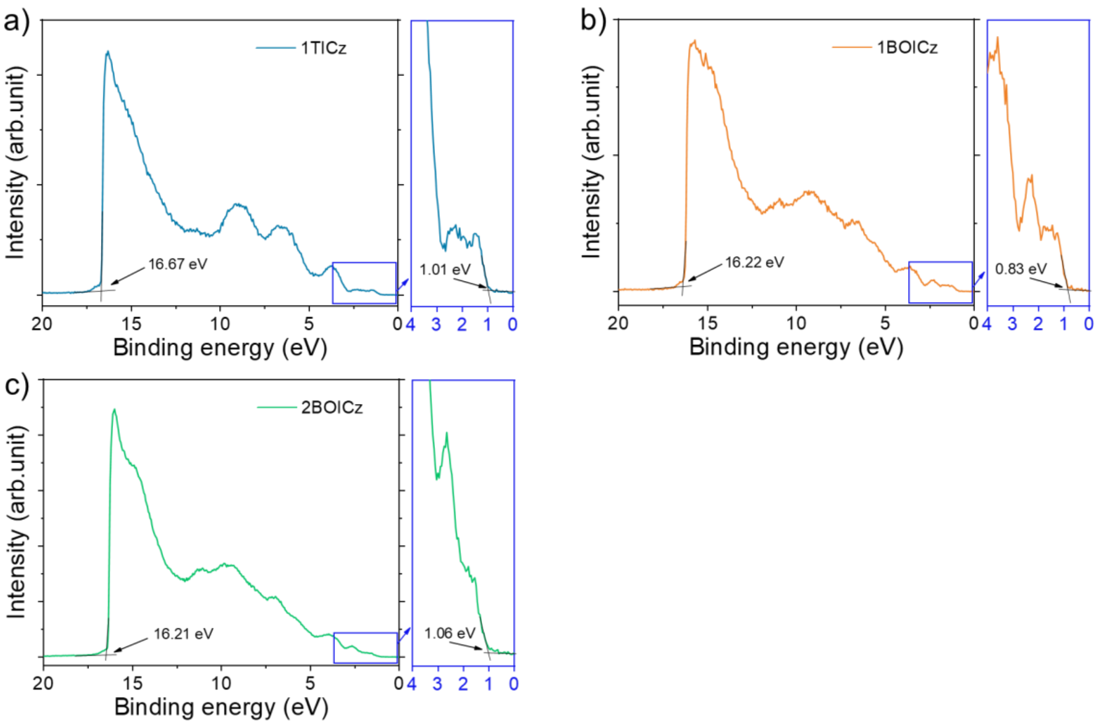

The work function of **1TICz**: WF=21.22−16.67=4.55 eV, the injection barrier relative to the substrate is 1.01 eV;
The work function of **1BOCz**: WF=21.22−16.22=5.00 eV, the injection barrier relative to the substrate is 0.89 eV;
The work function of **2BOCz**: WF=21.22−16.26=4.96 eV, the injection barrier relative to the substrate is 1.06 eV;
The ionization potential of **1TICz**: IP=21.22−(16.67−1.01)=5.56 eV
The ionization potential of **1BOCz**: IP=21.22−(16.22−0.89)=5.89 eV
The ionization potential of **2BOCz**: IP=21.22−(16.26−1.06)=6.02 eV
The above values of the ionization potential can be understood as the HOMO relative to the vacuum energy level.

Supplementary Fig. 6 The ultraviolet photoelectron spectra of the vacuum-deposited thin films for (a) 1TICz, (b) 1BOICz and (c) 2BOICz. The HOMO levels were determined to be −5.56 eV, −5.89 eV and −6.02 eV for 1TICz, 1BOICz and 2BOICz.

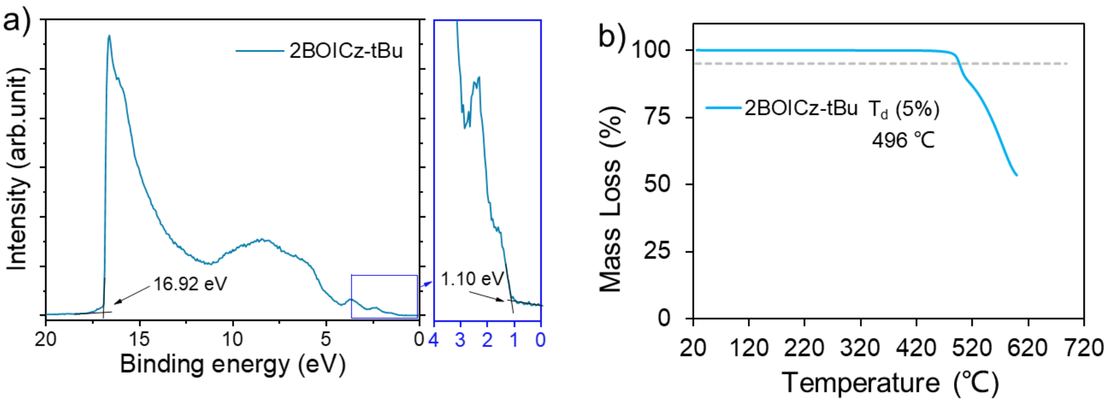

Supplementary Fig. 7 (a) The ultraviolet photoelectron spectra of the vacuum-deposited thin films for 2BOICz-tBu. The HOMO levels were determined to be −5.50 eV. The band energy is 2.98 eV, the LUMO energy level can be estimated to be −2.52 eV. (b) Decomposition temperature (T_d) with 5% weight loss for 2BOICz-tBu.

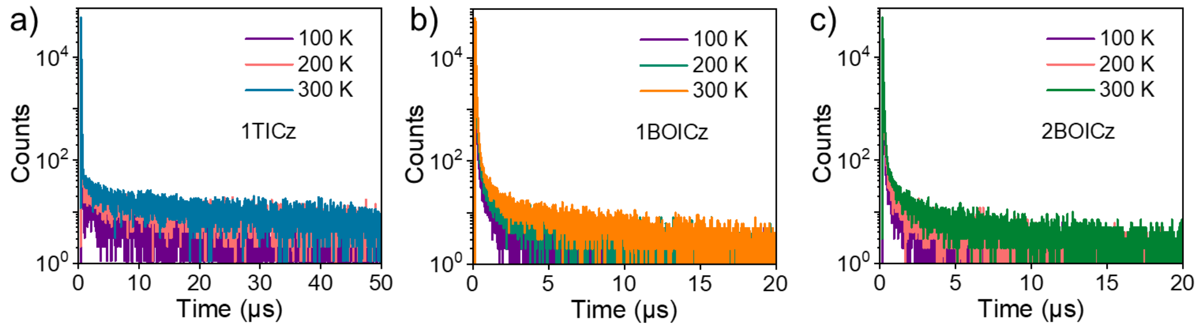

Supplementary Fig.8 Temperature-dependent transient PL decay spectra of 1TICz (a), 1BOICz (b) and 2BOICz (c) doped into the mCPBC films (20 wt%).

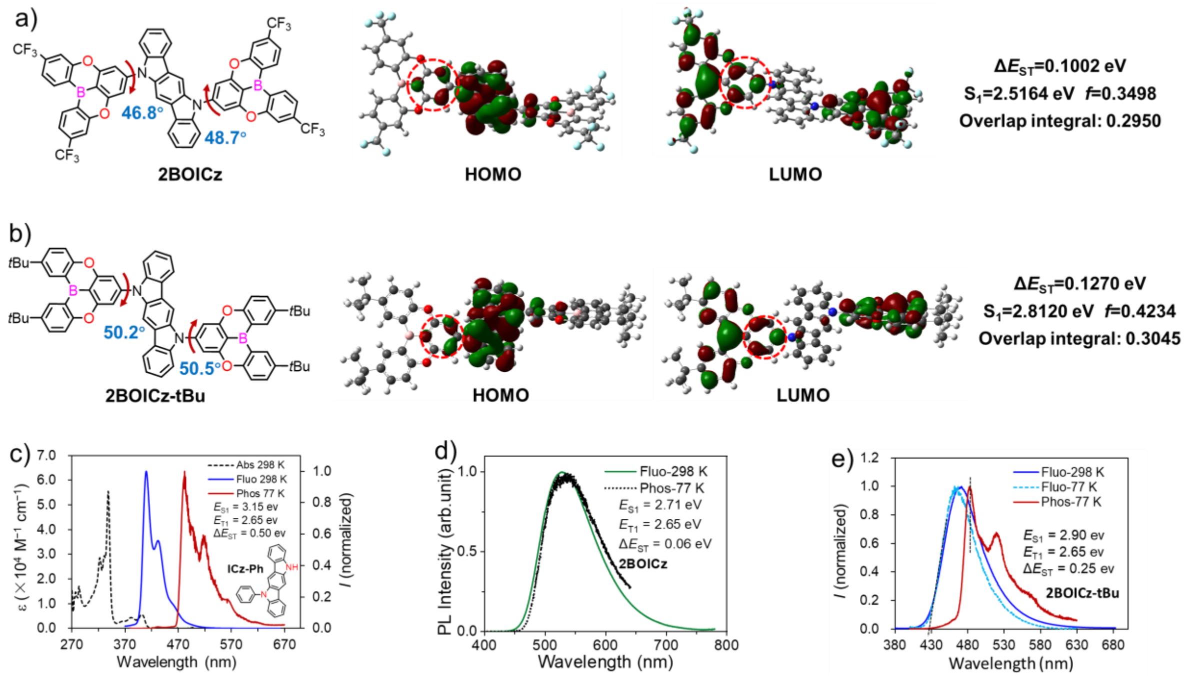

Supplementary Fig. 9 (a, b) The chemical structures of 2BOICz and 2BOICz-tBu, and the calculated values of the dihedral angle (*θ*), HOMO-LUMO distributions, singlet energy level (S_1) and oscillator strength (*f*). (c) UV-vis absorption (left axis) in toluene (10^{-5} M), normalized PL (298 K) and phosphorescence (77 K) spectra (right axis) of ICz-Ph in toluene. (d, e) Fluorescence (298 K or 77 K) and phosphorescence (77 K) spectra of doped films (20 wt% doped films).

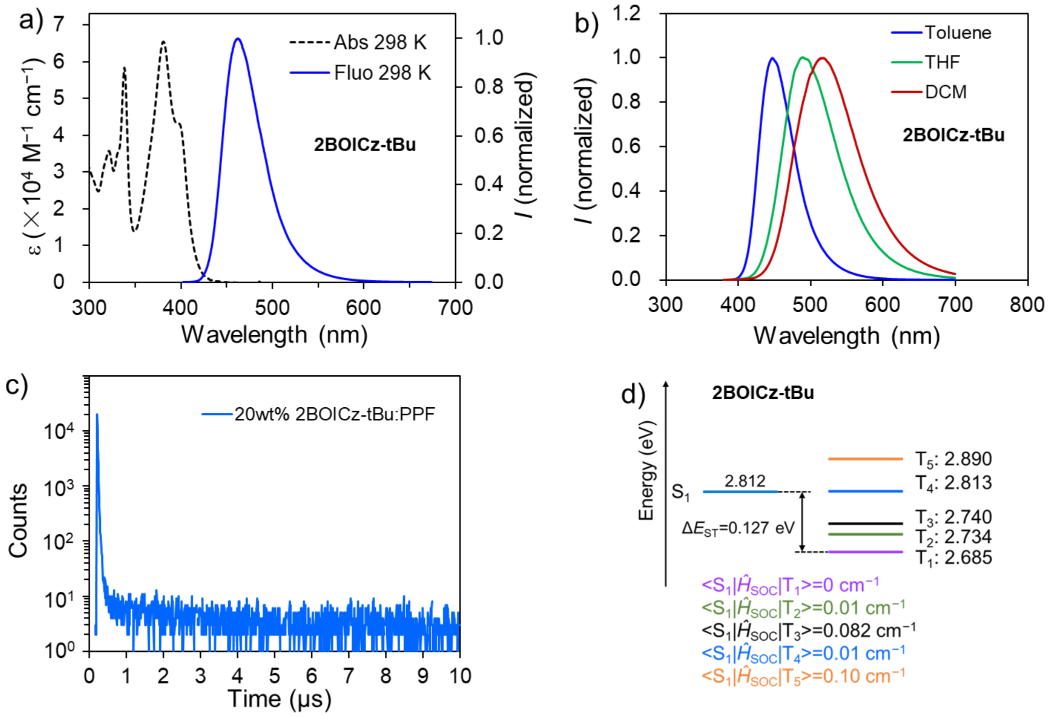

Supplementary Fig. 10 (a) UV-vis absorption (left axis) in toluene (10^{-5} M), and normalized PL spectra (right axis) of 2BOICz-tBu in toluene. (b) The solvent-dependent PL spectra. (c) Transient PL decay curve of 20 wt% 2BOICz-tBu doped film. (d) The spin-orbit coupling (SOC) matrix elements between S_1 and T_n states of 2BOICz-tBu were conducted at the B3LYP/6-31G(d,p) level.

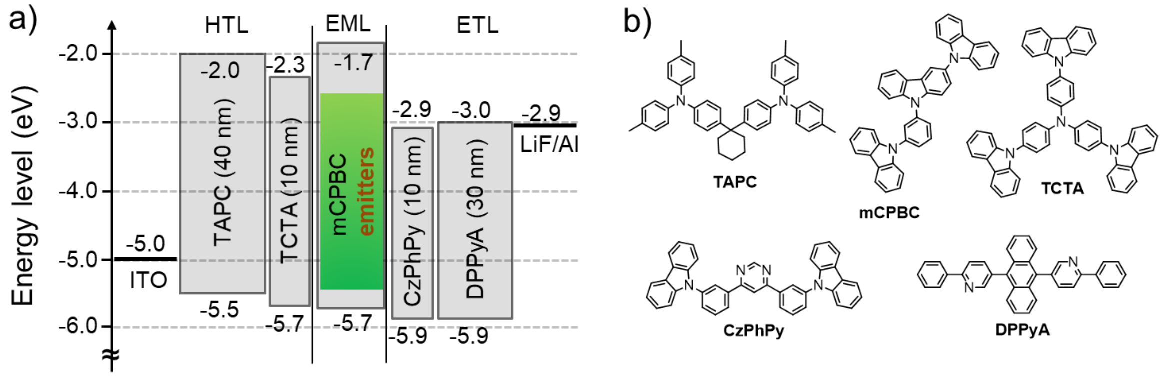

Supplementary Fig. 11 (a) Device architecture for TADF-OLEDs with energy levels of each layer material. (b) The major chemical structures of relevant materials.

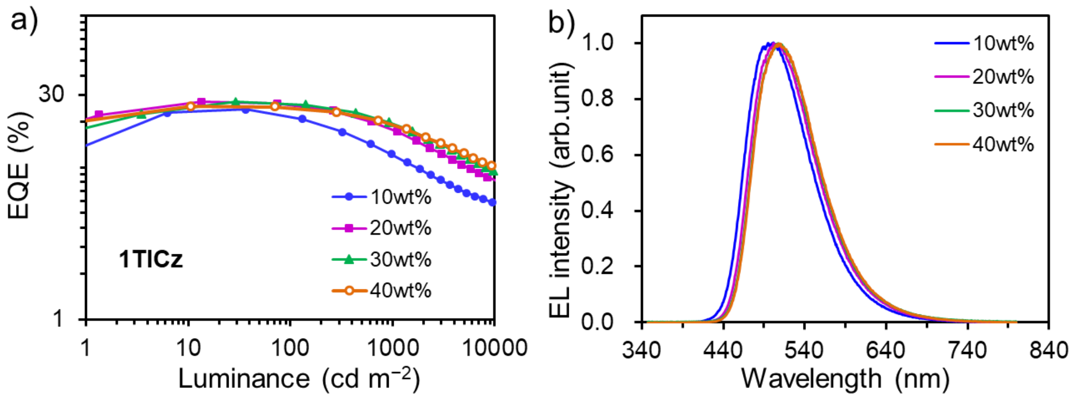

Supplementary Fig. 12 (a) External quantum efficiency (EQE) versus brightness for devices doped with 10 wt%, 20 wt%, 30 wt% and 40 wt% of 1TICz. (b) EL spectra of the devices under 1,000 cd m^{-2}.

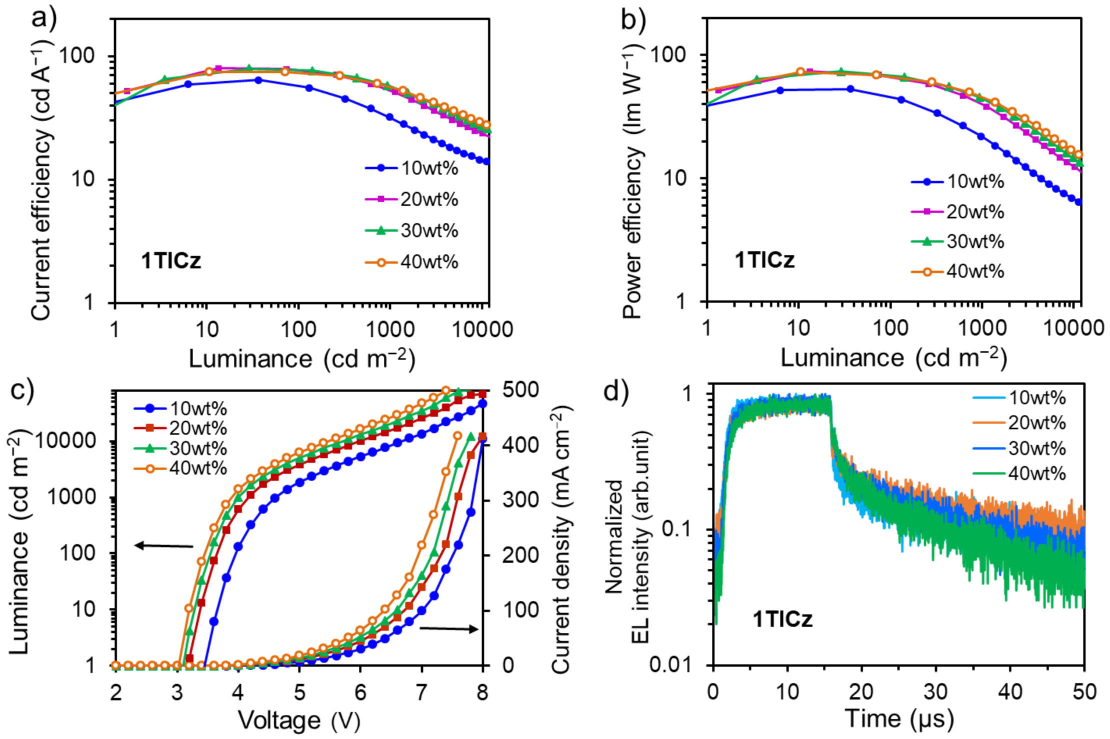

Supplementary Fig. 13 (a) Current efficiency (CE) and (b) power efficiency (PE) versus luminance (L), (c) Luminance–voltage–current density characteristics, and (d) EL transient spectra for the 10 wt%, 20 wt%, 30 wt% and 40 wt% doped devices based 1TICz.

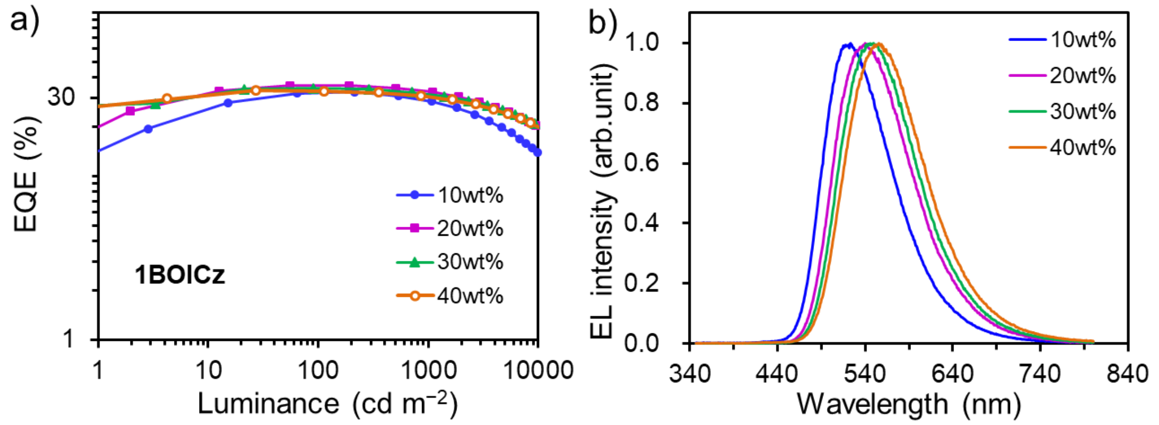

Supplementary Fig. 14 (a) External quantum efficiency (EQE) versus brightness for devices doped with 10 wt%, 20 wt%, 30 wt% and 40 wt% of 1BOICz. (b) EL spectra of the devices under 1,000 cd m^{-2}.

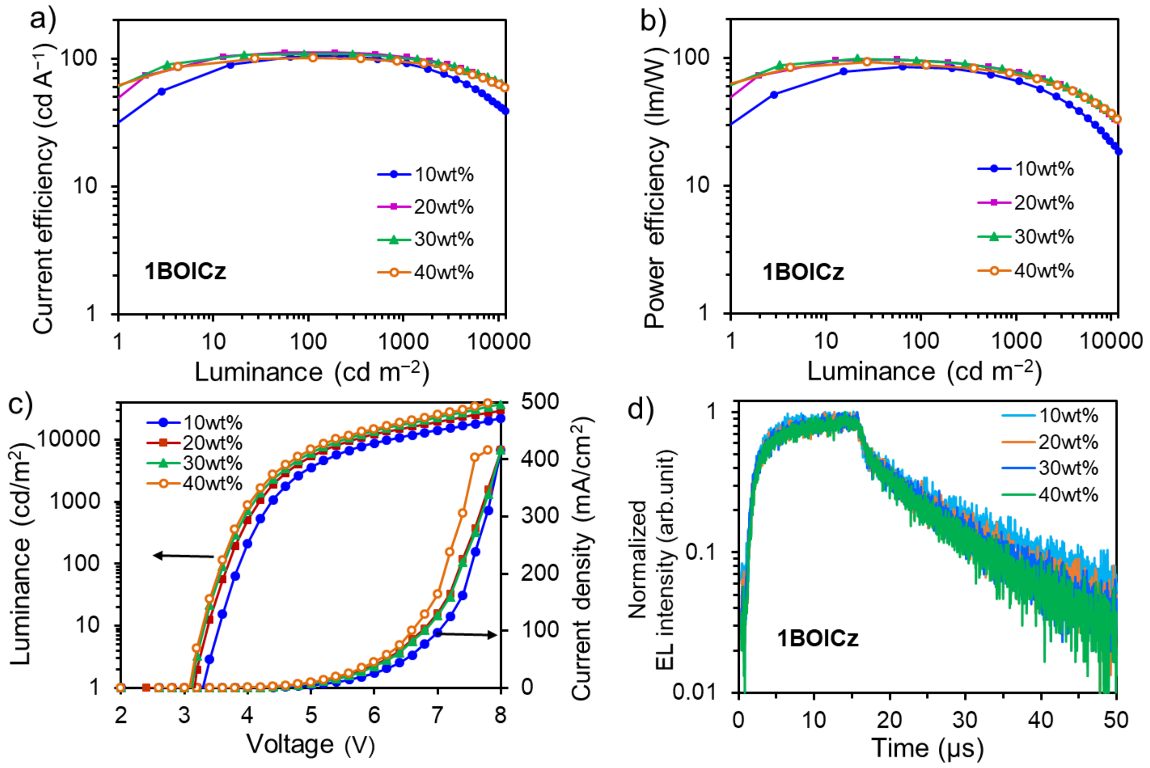

Supplementary Fig. 15 (a) Current efficiency (CE) and (b) power efficiency (PE) versus luminance (L), (c) Luminance–voltage–current density characteristics, and (d) EL transient spectra for the 10 wt%, 20 wt%, 30 wt% and 40 wt% doped devices based 1BOICz.

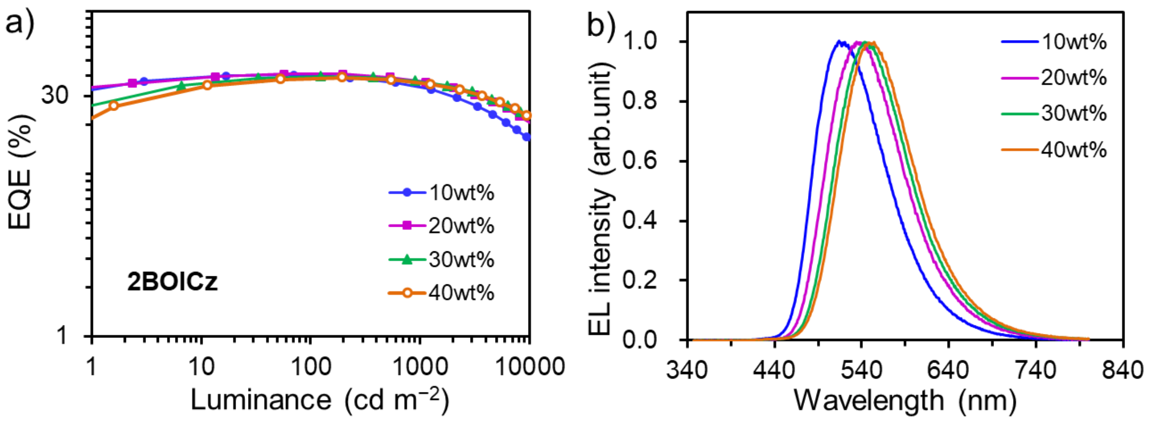

Supplementary Fig. 16 (a) External quantum efficiency (EQE) versus brightness for devices doped with 10 wt%, 20 wt%, 30 wt% and 40 wt% of 2BOICz. (b) EL spectra of the devices under 1,000 cd m^{-2}.

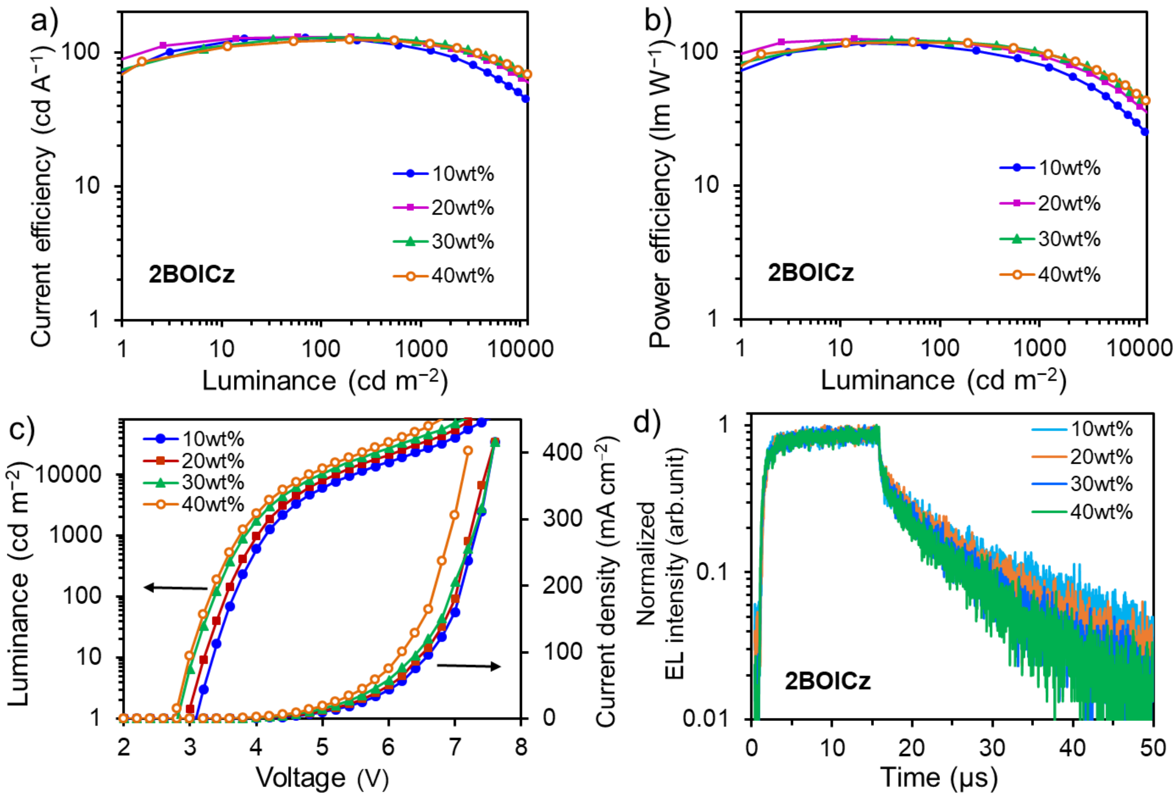

Supplementary Fig. 17 (a) Current efficiency (CE) and (b) power efficiency (PE) versus luminance (L), (c) Luminance–voltage–current density characteristics, and (d) EL transient spectra for the 10 wt%, 20 wt%, 30 wt% and 40 wt% doped devices based 2BOICz.

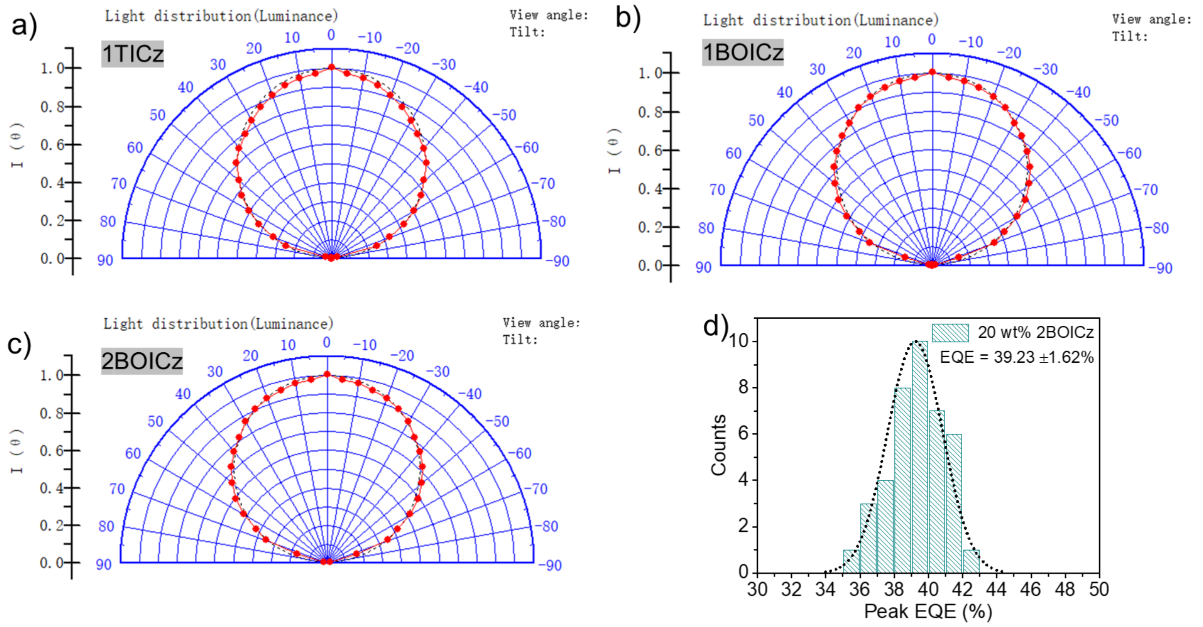

Supplementary Fig. 18 (a-c) Angle-dependent EL intensity of the TADF devices with 1TICz, 1BOICz and 2BOICz, compared to the Lambertian distribution, the correction coefficient is calculated to be 0.973, 0.978, 0.981, respectively. (d) The statistics of the peak EQEs based on 20 wt% doped 2BOICz measured from 40 devices, show an average peak EQE of 39.23% with a relative standard deviation of 1.62%.

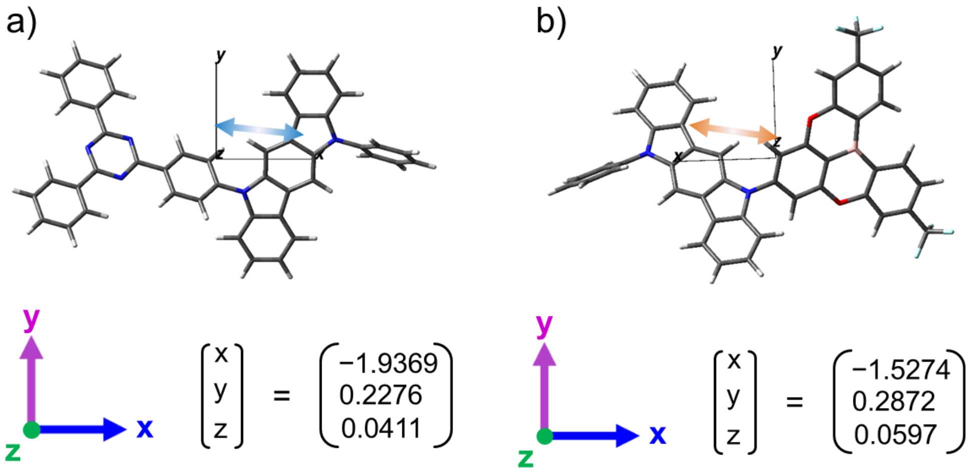

Supplementary Fig. 19 The direction of the calculated S_0–S_1 transition dipole moment (as indicated by the arrow) of (a) 1TICz and (b) 1BOICz based on optimized structure.

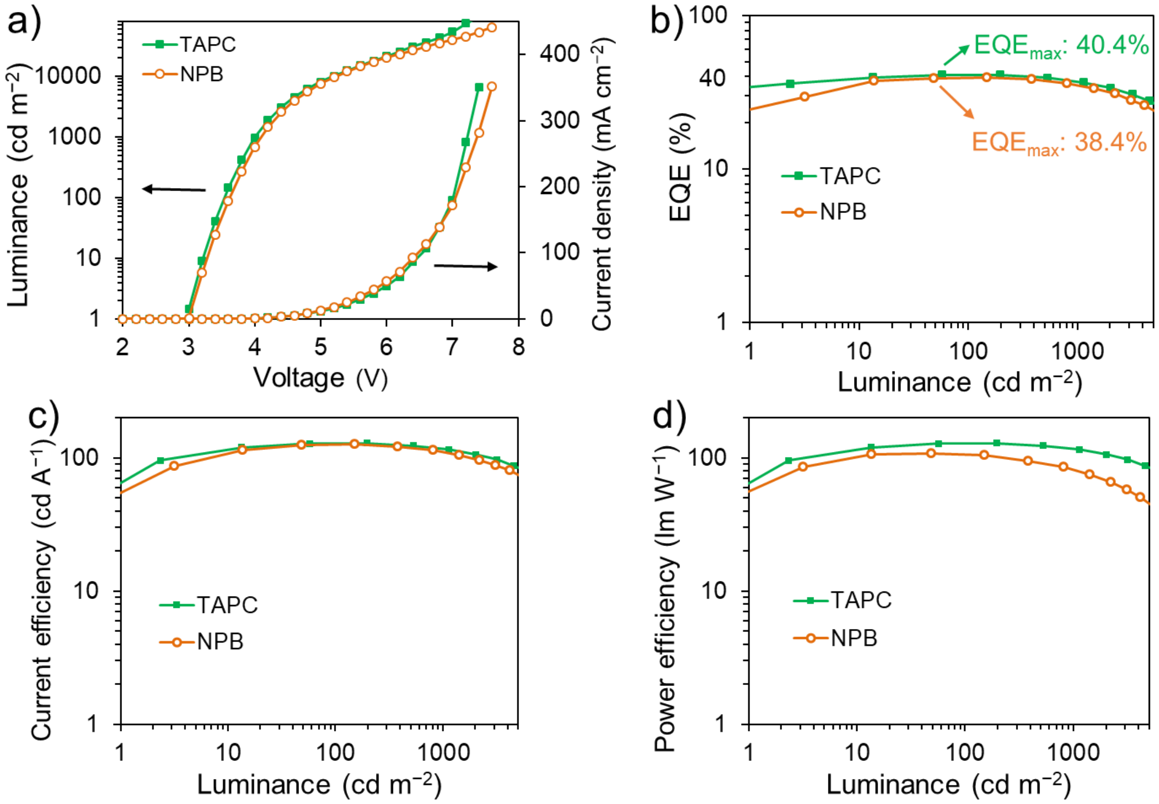

Supplementary Fig. 20 The effect of the hole transport layer (TAPC and NPB) on the device performance. The device structure: ITO/ TAPC or NPB (40 nm)/ TCTA (10 nm)/ mCPBC: 20 wt% 2BOICz (24 nm)/ CzPhPy (10 nm)/ DPPyA (30 nm)/ LiF (0.5 nm)/ Al (150 nm). (a) Luminance-voltage-current density characteristics. (b) External quantum efficiency (EQE), (c) current efficiency (CE) and (d) power efficiency (PE) versus luminance.

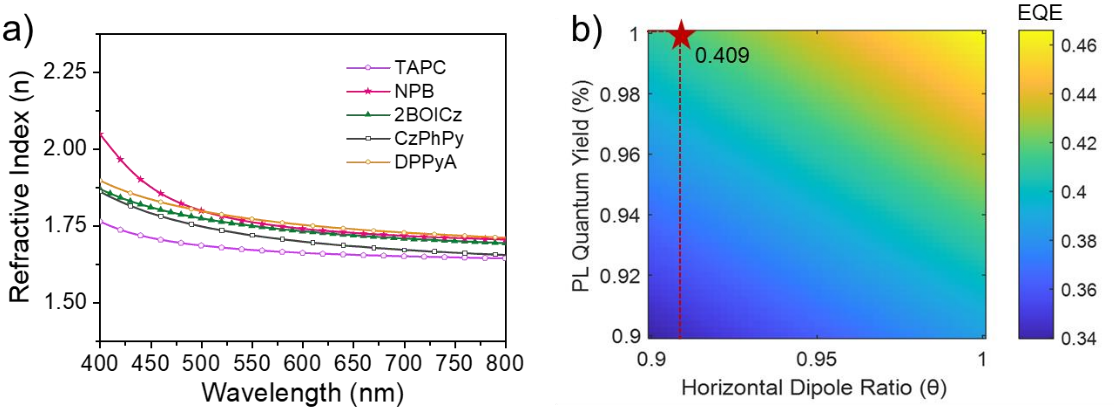

Supplementary Fig. 21 (a) The refractive index values of the functional layer and emitting layer (mCPBC: 20 wt% 2BOICz). (b) Theoretically calculated TADF device EQE value with respect to PLQY and horizontal dipole ratio.

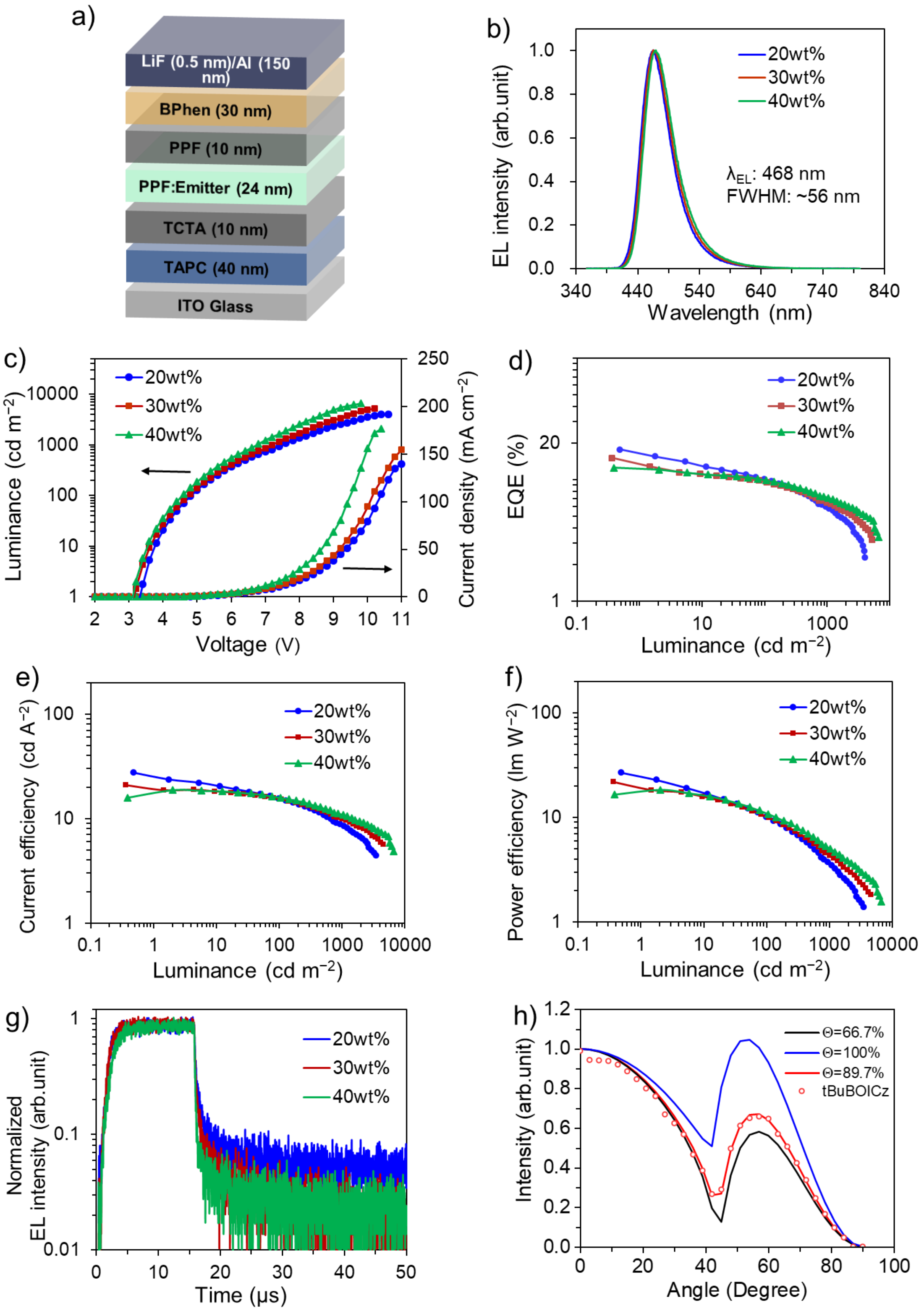

Supplementary Fig. 22 (a) Device architecture for 2BOICz-tBu based TADF-OLEDs. (b) EL spectra of the devices under 1,000 cd m^{-2}. (c) Luminance-voltage-current density characteristics. (d) External quantum efficiency (EQE), (e) current efficiency (CE) and (f) power efficiency (PE) versus luminance. (g) EL transient spectra for the 20 wt%, 30 wt% and 40 wt% 2BOICz-tBu doped devices. (h) Angle-dependent PL spectra of 20 wt% 2BOICz-tBu in PPF host.

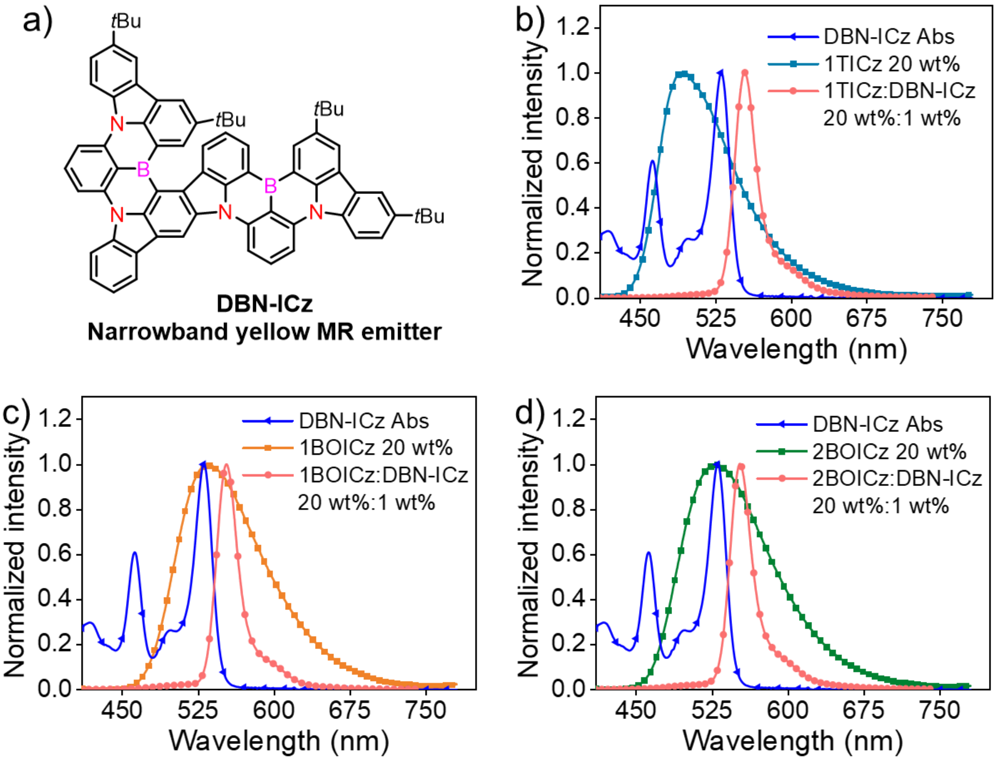

Supplementary Fig. 23 (a) The chemical structure of narrowband yellow MR emitter DBN-ICz. (b, c, and d) UV-Vis absorption of DBN-ICz in toluene with a concentration of 10^{-5} mol^{-1}, the PL spectra of 20 wt% 1TICz (or 1BOICz, 2BOICz) and 20 wt% 1TICz (or 1BOICz, 2BOICz): 1 wt% DBN-ICz doped in mCPBC.

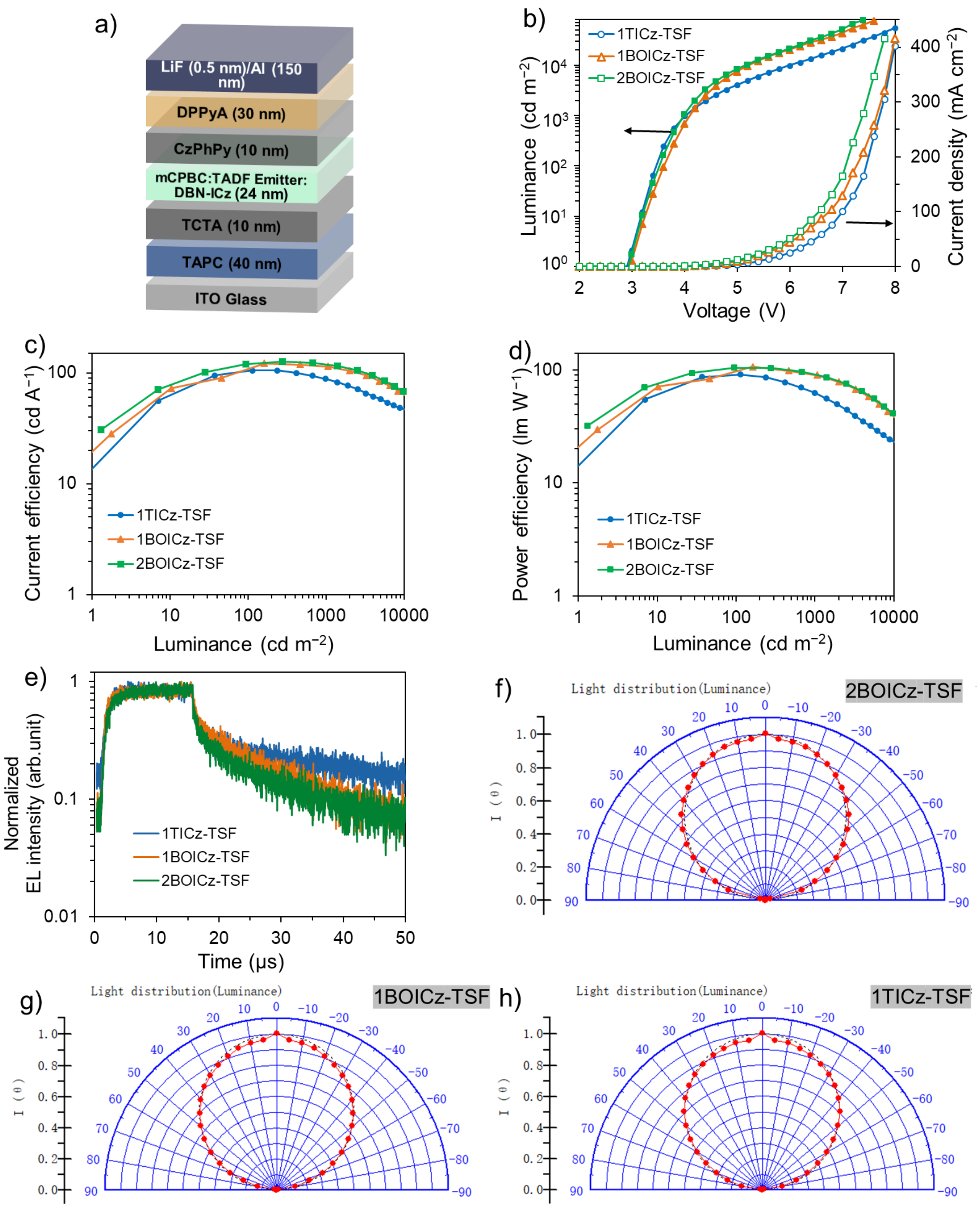

Supplementary Fig. 24 (a) Device architecture for TSF-OLEDs based 1TICz, 1BOICz and 2BOICz. (b) Luminance-voltage-current density characteristics. (c) The current efficiency (CE) and (d) power efficiency (PE) versus luminance. (e) EL transient spectra for the TSF-OLEDs. (f-h) Angle-dependent EL intensities of the TSF-OLEDs with 2BOICz, 1BOICz and 1TICz, compared to the Lambertian distribution, the correction coefficient is calculated to be 0.980, 0.972, 0.970, respectively.

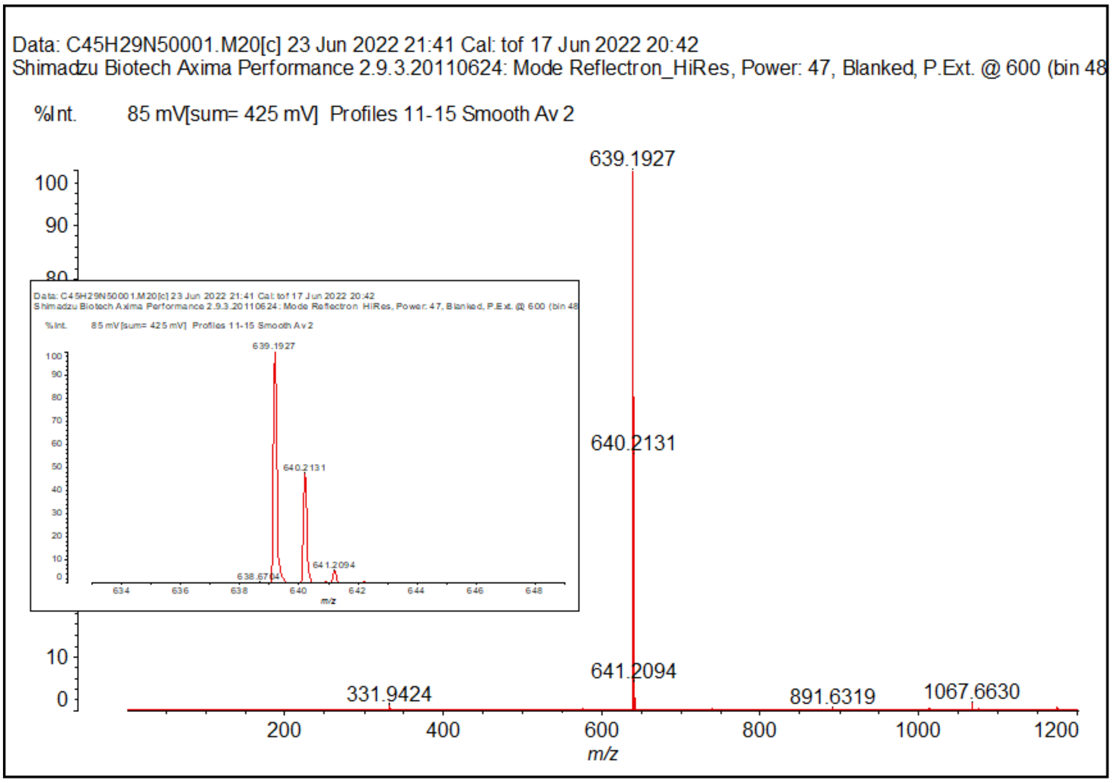

Supplementary Fig. 25 Mass spectrometry of **1TICz**.

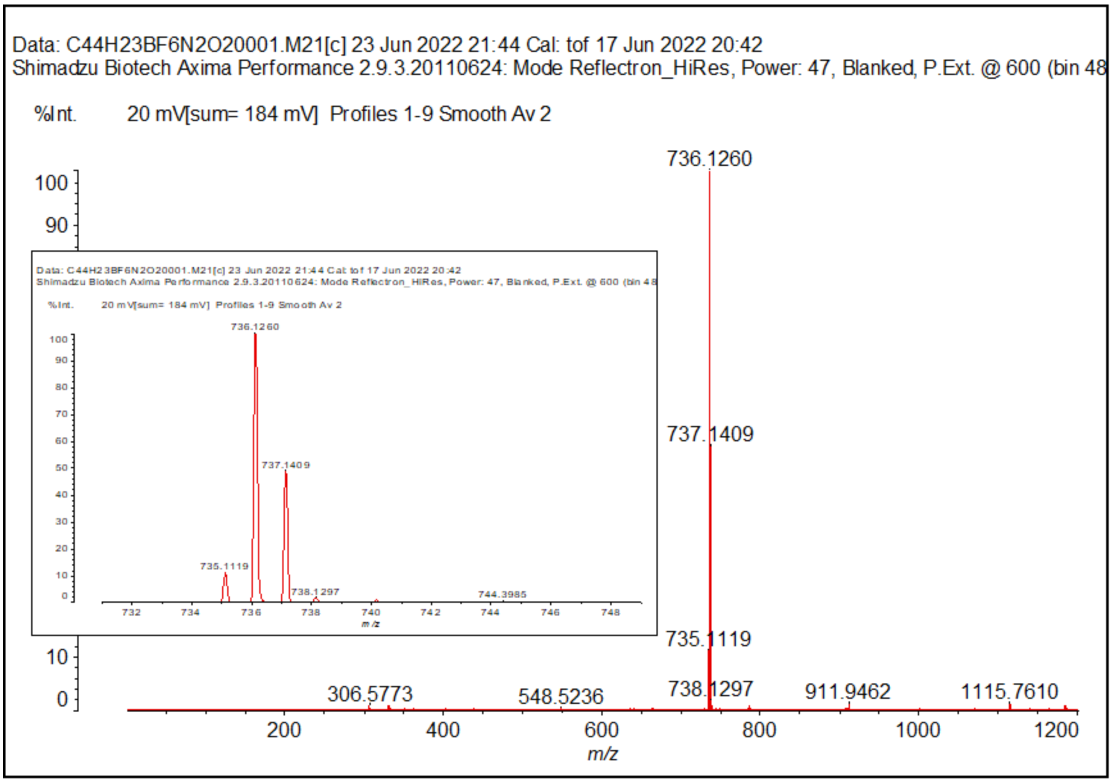

Supplementary Fig. 26 Mass spectrometry of **1BOICz**.

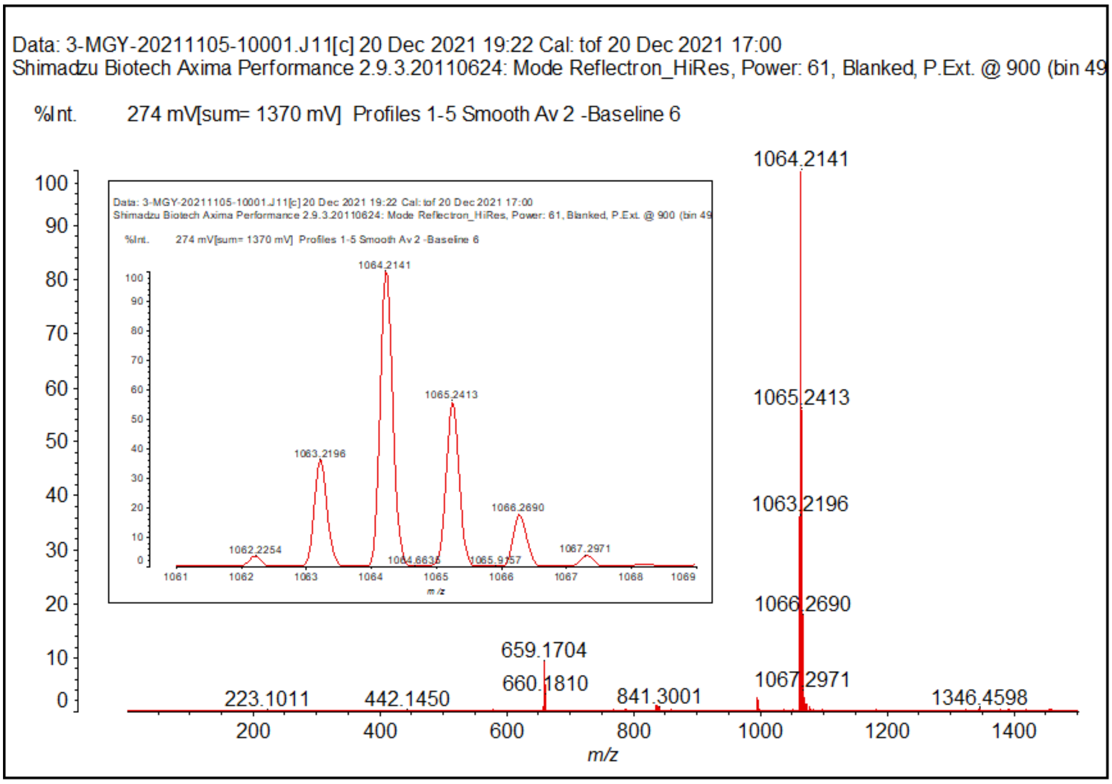

Supplementary Fig. 27 Mass spectrometry of **2BOICz**.

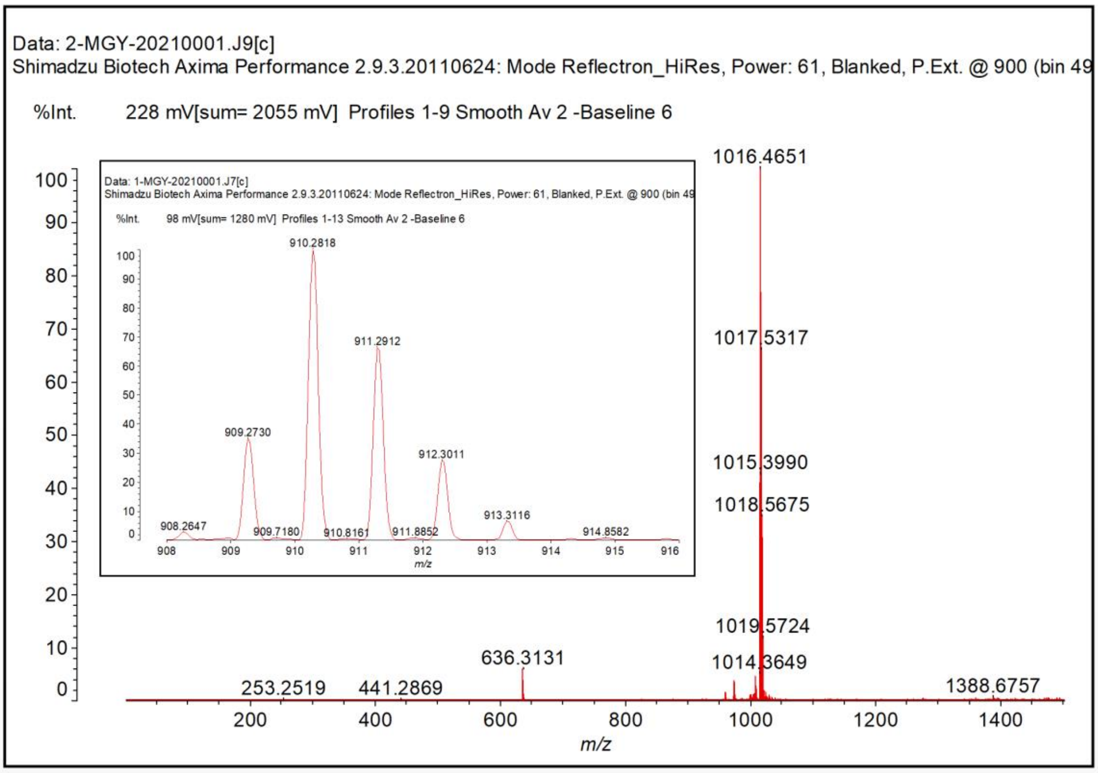

Supplementary Fig. 28 Mass spectrometry of **2BOICz-tBu**.

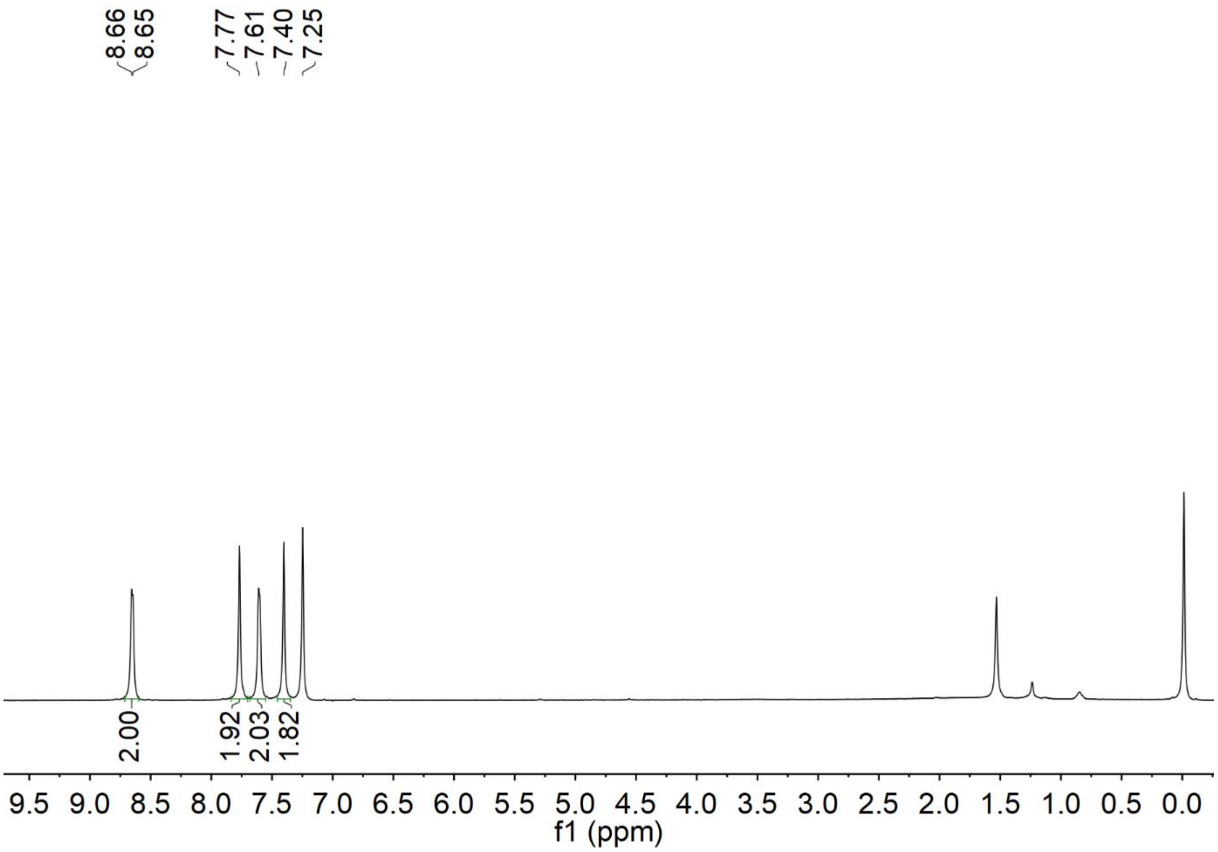

Supplementary Fig. 29 ^{1}H NMR spectrum of **BO-Br** in $CDCl_3$.

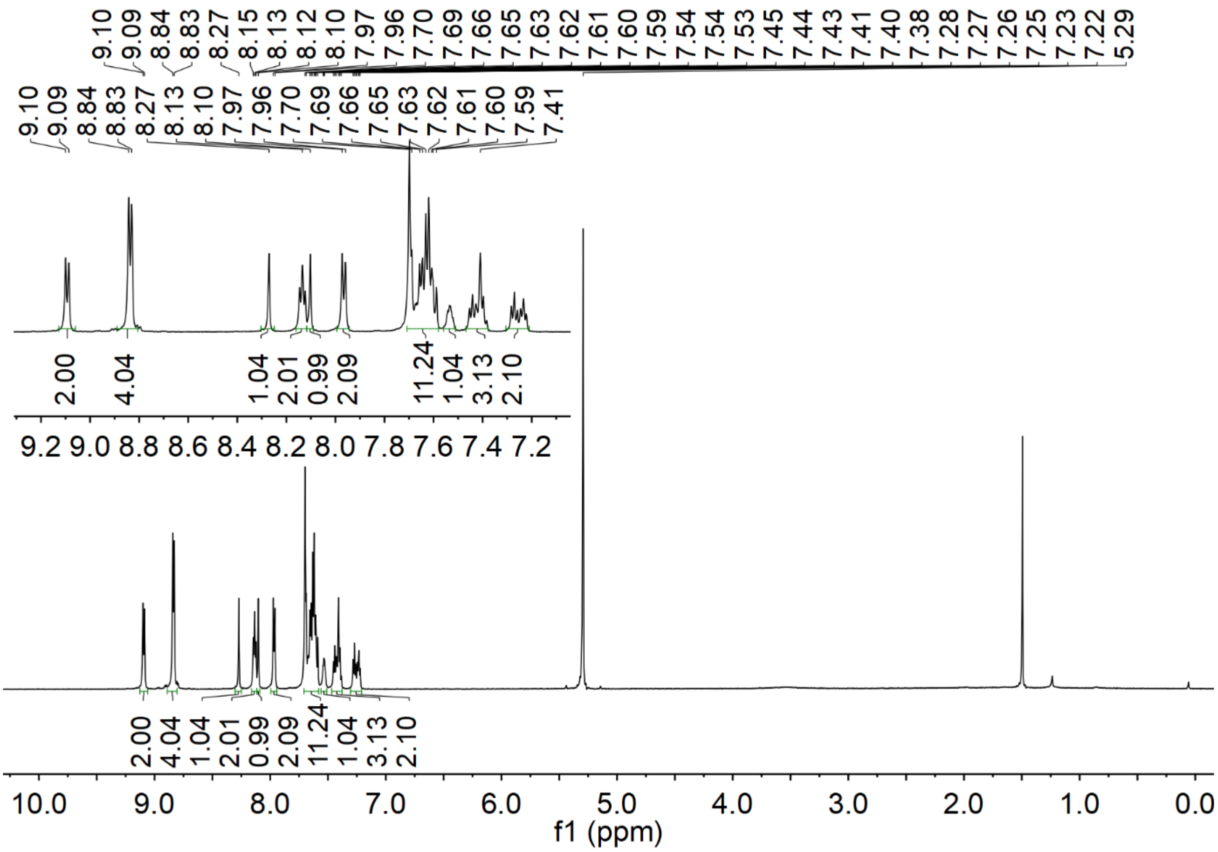

Supplementary Fig. 30 ^{1}H NMR spectrum of **1TICz** in CD_2Cl_2.

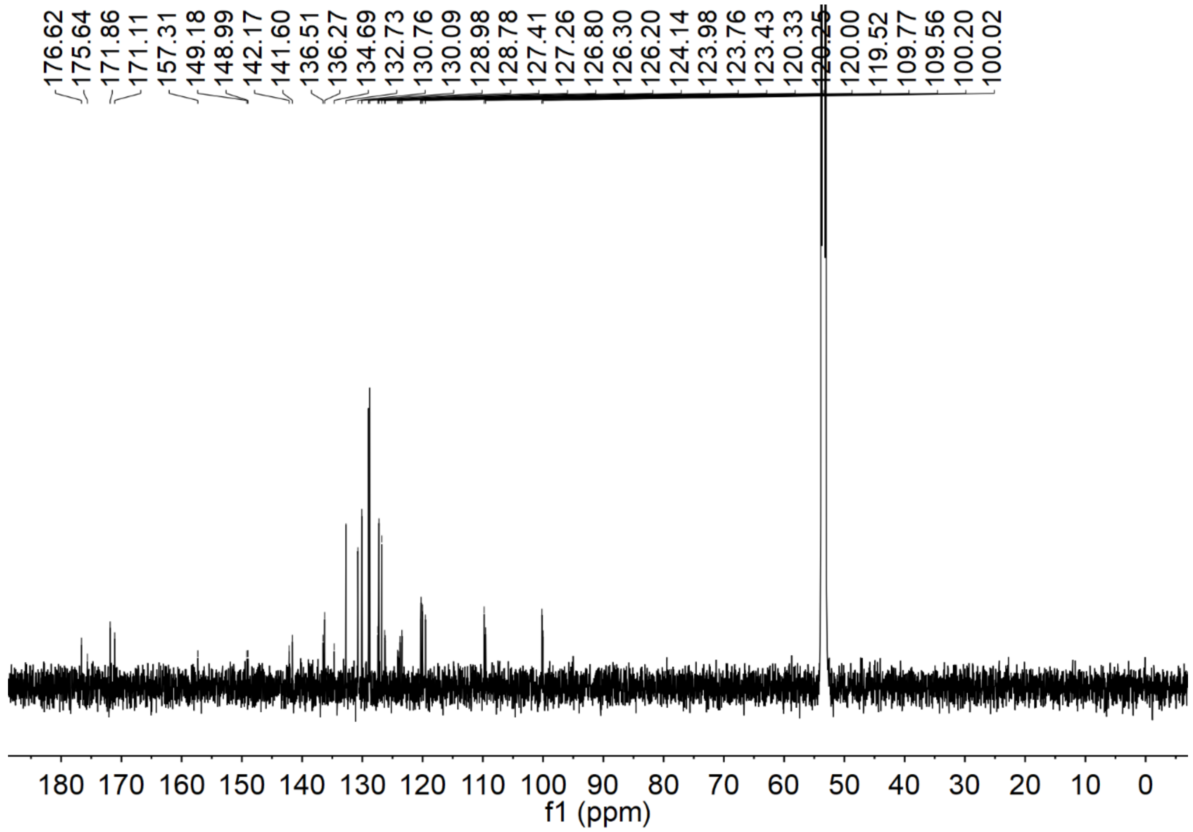

Supplementary Fig. 31 ^{13}C NMR spectrum of **1TICz** in CD_2Cl_2.

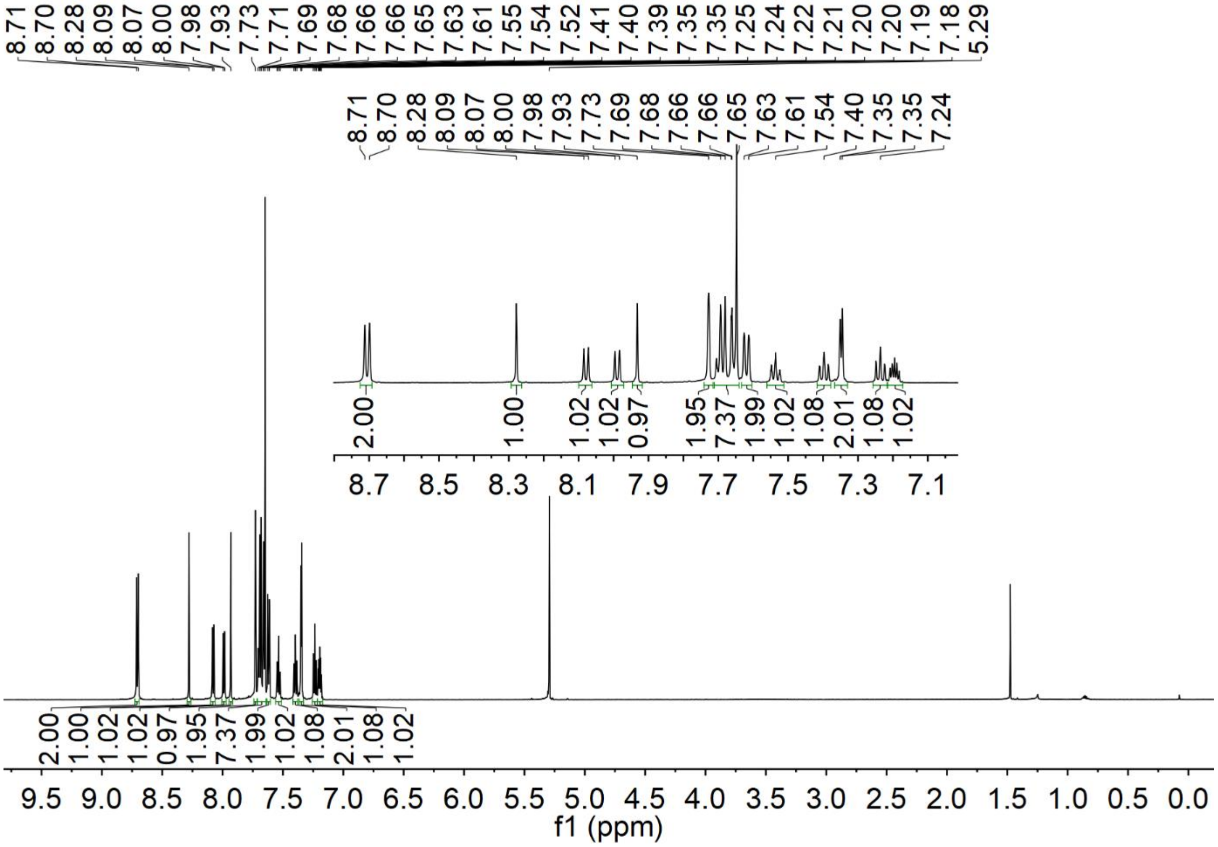

Supplementary Fig. 32 ^{1}H NMR spectrum of **1BOICz** in CD_2Cl_2.

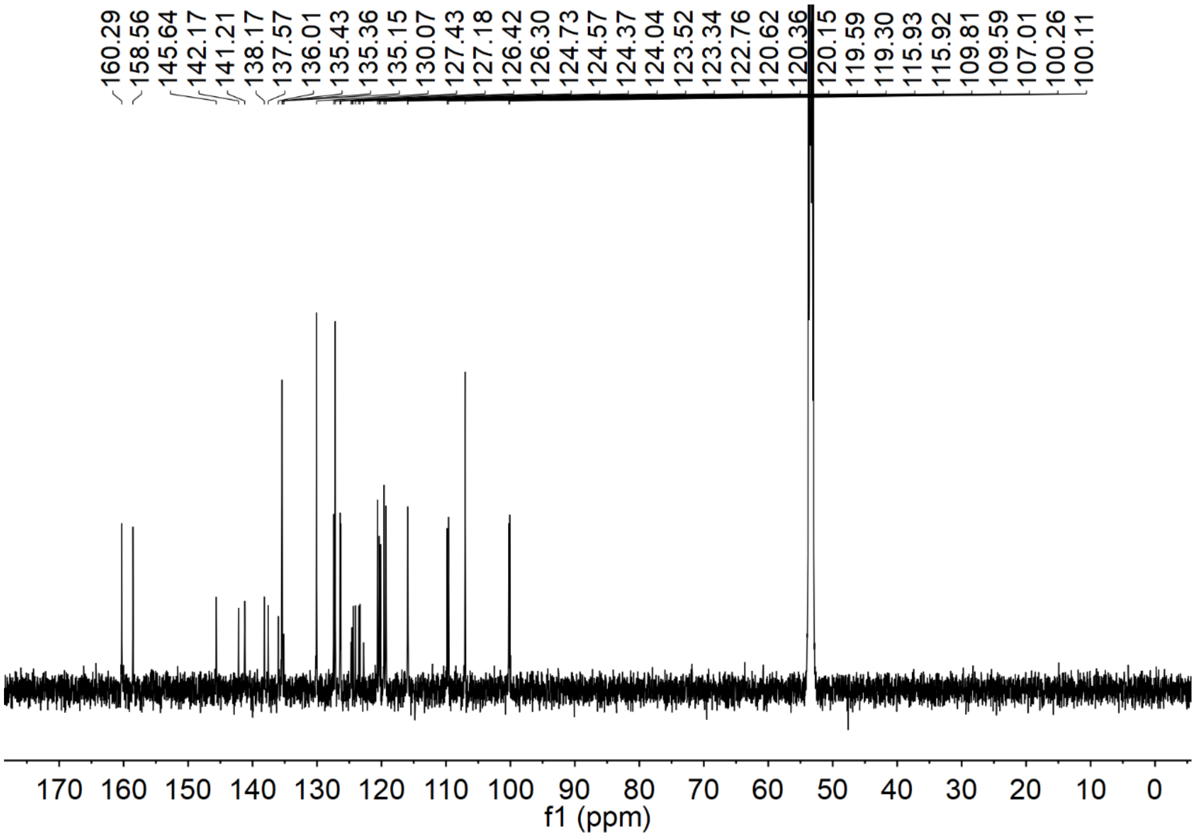

Supplementary Fig. 33 ^{13}C NMR spectrum of **1BOICz** in CD_2Cl_2.

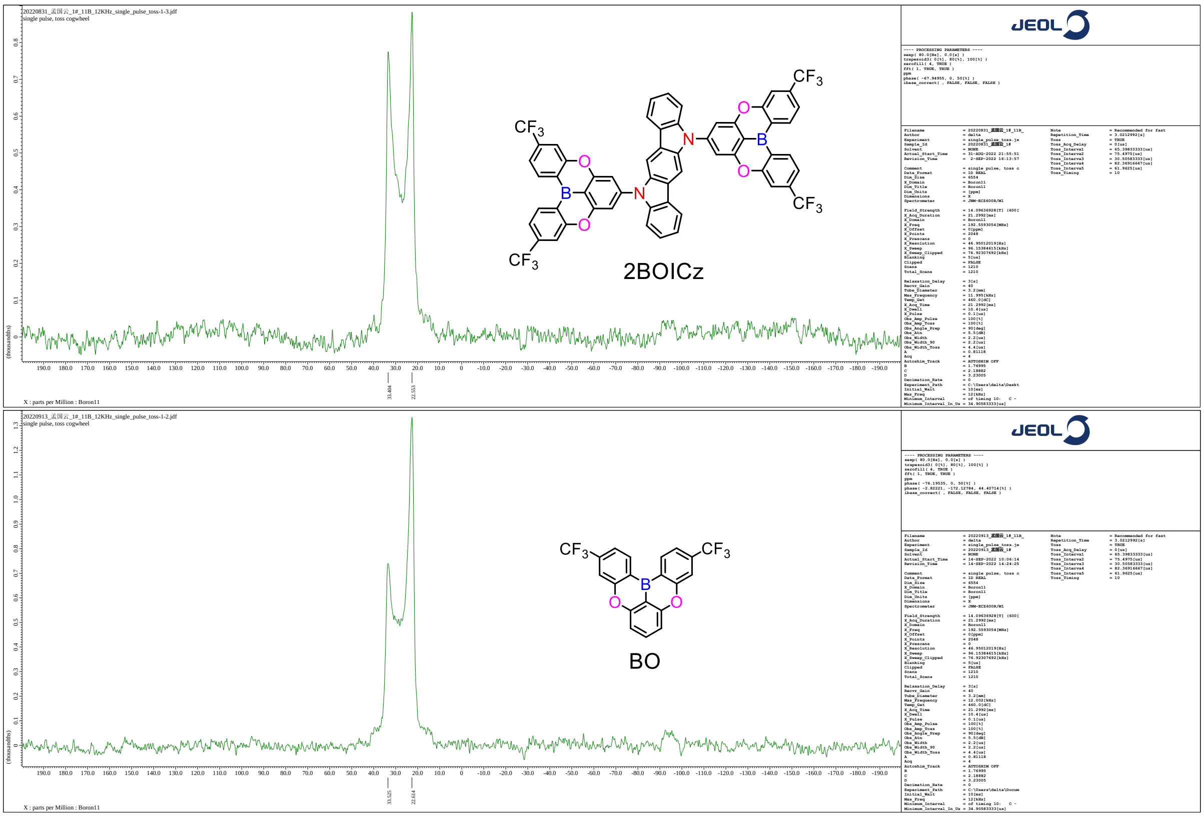

Supplementary Fig. 34 ^{11}B solid-state NMR spectra of **2BOICz** and the parent core **CF_3-BO**.

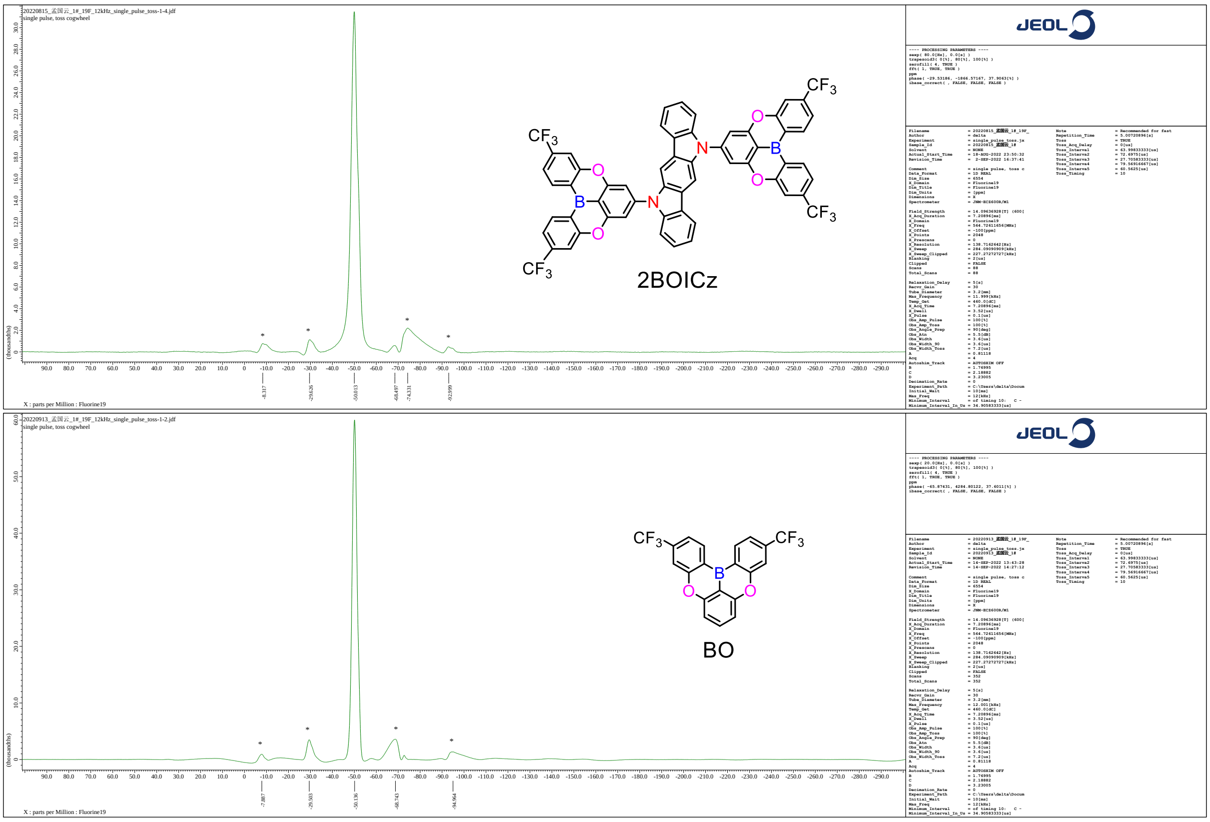

Supplementary Fig. 35 ^{19}F solid-state NMR spectra of **2BOICz** and the parent core **CF$_3$-BO**. The * represents rotating sidebands.

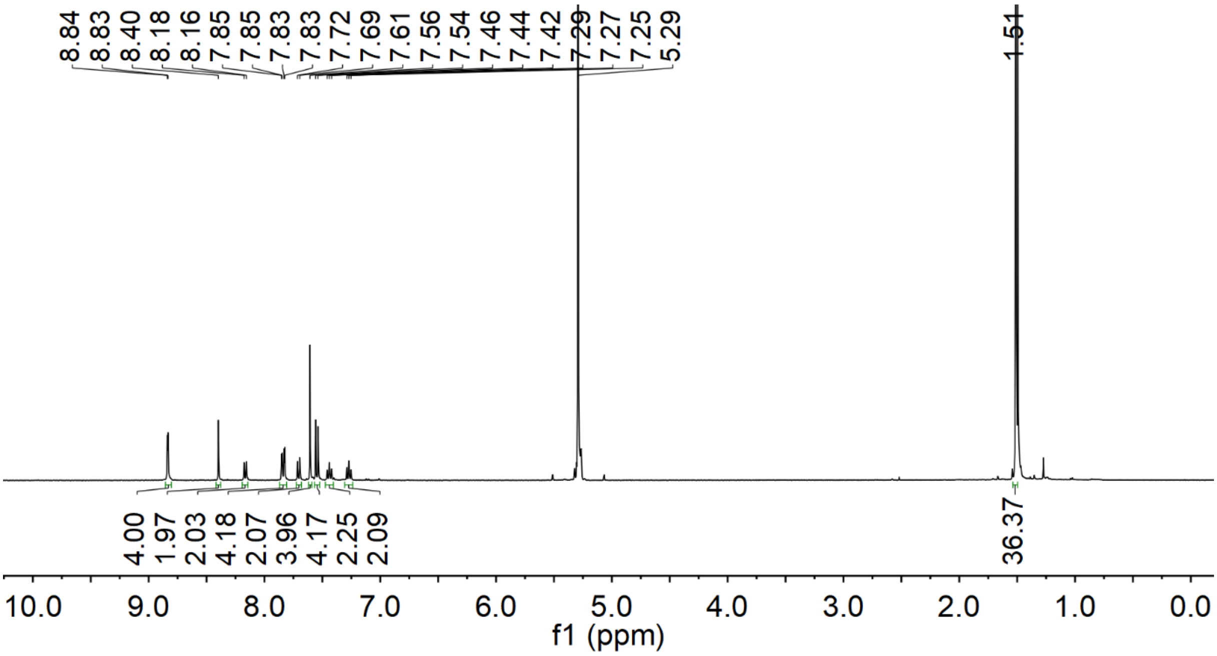

Supplementary Fig. 36 ^{1}H NMR spectrum of **2BOICz-tBu** in CD_2Cl_2.

2. Supplementary Tables

Supplementary Table 1 Summary of TD-DFT calculation for 1TICz, 1BOICz and 2BOICz at the S_0 structures at the B3LYP/6-31G(d, p) level.

Compound	Optimized Structure	Transition	Wavelength (nm)	Energy (eV)	Oscillator Strength
1TICz	S_0	$S_0 \rightarrow S_1$	469.09	2.6434	0.2361
		$S_0 \rightarrow T_1$	506.08	2.4502	0
1BOICz	S_0	$S_0 \rightarrow S_1$	513.84	2.4129	0.1426
		$S_0 \rightarrow T_1$	537.21	2.3075	0
2BOICz	S_0	$S_0 \rightarrow S_1$	492.70	2.5164	0.3498
		$S_0 \rightarrow T_1$	513.13	2.4162	0

Supplementary Table 2 Photophysical properties of emitters.

Compound	λ_{PL} [nm][a]	FWHM [nm][b]	Φ_{PL} [%][c]	E_g [eV][d]	HOMO[e]/LUMO [eV]
1TICz	483	84	90	2.80	−5.56/−2.76
1BOICz	527	85	90	2.63	−5.89/−3.26
2BOICz	518	83	89[f]	2.64	−6.02/−3.38

[a] Measured in toluene (10^{-5} M) at room temperature. [b] Full-width at half-maximum of the PL spectrum given in wavelength. [c] Absolute PL quantum yield measured in degassed toluene using an integrating sphere. [d] Estimated from absorption edges of UV–vis spectra. [e] Determined from ultraviolet photoelectron spectra, LUMO was calculated by E_g and HOMO. [f] The slightly lower Φ_{PL} value is probably due to the more significant rotation of two acceptors in 2BOICz.

Supplementary Table 3 Summary of the EL performance of 1TICz based-devices.

Doping concentration	λ_{EL} [a] [nm]	V_{on}[b] [V]	L_{max} [c] [cd m^{-2}]	$CE_{max/1,000/5,000}$ [d] [cd A^{-1}]	$PE_{max/1,000/5,000}$ [e] [lm W^{-1}]	$EQE_{max/1,000/5,000}$ [f] [%]	CIE (x,y) [g]
10 wt%	495	3.5	46,790	64.3/32.1/17.2	53.1/21.9/9.0	22.9/12.2/6.8	(0.222, 0.446)
20 wt%	504	3.1	68,910	80.0/51.1/30.6	71.7/37.1/18.0	26.1/16.7/10.1	(0.247, 0.499)
30 wt%	507	3.0	82,100	79.6/58.0/35.0	73.5/45.5/22.0	26.9/17.3/12.1	(0.258, 0.522)
40 wt%	508	3.0	84,950	74.8/52.4/35.8	69.1/41.1/23.4	25.1/17.7/12.3	(0.265, 0.527)

[a] EL peak wavelength; [b] Turn-on voltage (V_{on}); [c] Maximum luminescence (L); [d] Maximum current efficiency (CE), value at 1,000 and 5,000 cd cm^{-2}; [e] Maximum power efficiency (PE), value at 1,000 and 5,000 cd cm^{-2}; [f] Maximum external quantum efficiency (EQE), value at 1,000 and 5,000 cd cm^{-2}; [g] CIE coordinates at 1,000 cd cm^{-2}.

Supplementary Table 4 Summary of the EL performance of 1BOICz based-devices.

Doping concentration	λ_{EL} [a] [nm]	V_{on} [b] [V]	L_{max} [c] [cd m^{-2}]	$CE_{max/1,000/5,000}$ [d] [cd A^{-1}]	$PE_{max/1,000/5,000}$ [e] [lm W^{-1}]	$EQE_{max/1,000/5,000}$ [f] [%]	CIE (x,y) [g]
10 wt%	524	3.3	62,250	105.4/92.2/63.2	85.4/65.8/38.2	32.6/28.7/19.9	(0.314, 0.572)
20 wt%	534	3.1	77,310	112.3/103.6/78.4	95.5/76.0/48.2	34.6/31.7/24.2	(0.381, 0.566)
30 wt%	550	3.1	80,770	109.0/98.8/81.4	98.9/73.9/53.3	34.3/30.7/25.0	(0.403, 0.559)
40 wt%	556	3.1	84,390	100.9/96.1/74.9	92.6/75.5/49.1	33.3/29.2/23.9	(0.428, 0.544)

[a] EL peak wavelength; [b] Turn-on voltage (V_{on}); [c] Maximum luminescence (L); [d] Maximum current efficiency (CE), value at 1,000 and 5,000 cd cm^{-2}; [e] Maximum power efficiency (PE), value at 1,000 and 5,000 cd cm^{-2}; [f] Maximum external quantum efficiency (EQE), value at 1,000 and 5,000 cd cm^{-2}; [g] CIE coordinates at 1,000 cd cm^{-2}.

Supplementary Table 5 Summary of the EL performance of 2BOICz based-devices.

Doping concentration	λ_{EL} [a] [nm]	V_{on} [b] [V]	L_{max} [c] [cd m^{-2}]	$CE_{max/1,000/5,000}$ [d] [cd A^{-1}]	$PE_{max/1,000/5,000}$ [e] [lm W^{-1}]	$EQE_{max/1,000/5,000}$ [f] [%]	CIE (x,y) [g]
10 wt%	521	3.1	91,410	128.4/102.9/71.2	116.6/76.9/46.6	39.8/32.4/22.8	(0.297, 0.554)
20 wt%	528	3.0	106,800	130.4/116.3/87.3	122.4/89.6/58.4	40.4/35.9/27.1	(0.383, 0.550)
30 wt%	539	2.8	129,100	129.0/122.4/90.2	122.1/101.2/60.6	39.7/35.0/27.1	(0.389, 0.564)
40 wt%	554	2.7	133,100	124.8/115.2/88.7	118.5/95.2/63.3	38.9/35.6/27.3	(0.406, 0.556)

[a] EL peak wavelength; [b] Turn-on voltage (V_{on}); [c] Maximum luminescence (L); [d] Maximum current efficiency (CE), value at 1,000 and 5,000 cd cm^{-2}; [e] Maximum power efficiency (PE), value at 1,000 and 5,000 cd cm^{-2}; [f] Maximum external quantum efficiency (EQE), value at 1,000 and 5,000 cd cm^{-2}; [g] CIE coordinates at 1,000 cd cm^{-2}.

Supplementary Table 6 Summary of the EL performance of 2BOICz-tBu based devices.

Doping concentration	λ_{EL} [a] [nm]	V_{on} [b] [V]	L_{max} [c] [cd m^{-2}]	$CE_{max/100}$ [d] [cd A^{-1}]	$PE_{max/100}$ [e] [lm W^{-1}]	$EQE_{max/100}$ [f] [%]	CIE (x,y) [g]
20 wt%	467	3.3	3987	27.5/15.6	27.0/10.2	17.7/10.1	(0.157, 0.201)
30 wt%	468	3.0	5200	21.0/15.3	22.0/10.0	15.1/9.8	(0.155, 0.214)
40 wt%	469	2.9	6589	18.8/15.9	18.5/10.8	12.5/9.8	(0.156, 0.212)

[a] EL peak wavelength; [b] Turn-on voltage (V_{on}); [c] Maximum luminescence (L); [d] Maximum current efficiency (CE), value at 100 cd cm^{-2}; [e] Maximum power efficiency (PE), value at 100 cd cm^{-2}; [f] Maximum external quantum efficiency (EQE), value at 100 cd cm^{-2}; [g] CIE coordinates at 1,000 cd cm^{-2}.

Supplementary Table 7 Summary of the reported TADF emitters with nearly 40% EQE_{max} values in OLED.

TADF materials	Refs	Φ_{PL} (%)	$\Theta_{//}$ (%)	λ_{EL} (nm)	$EQE_{max,100,1000}$ (%)
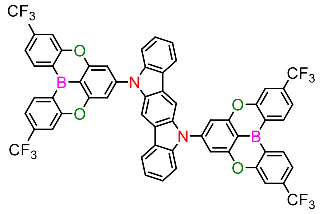 2BOICz	**This work**	**100%**	**92**	**528**	**41.2/41.1/36.6**
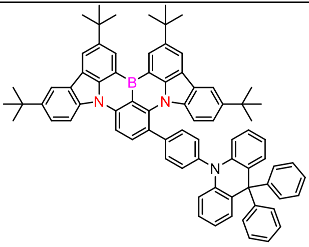 m-DPAcP-BNCz	1	97	86	496	42.0/36.7/17.5
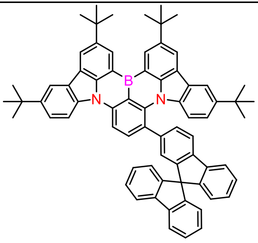 m-SF-BNCz	1	98	87	496	41.1/37.0/17.9
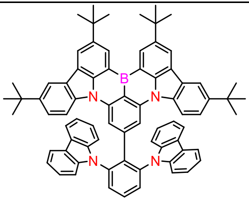 BN-CP1	2	98	93	496	40.0/34.0/18.5
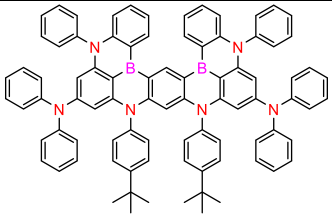 t-Bu-ν-DABNA HF	3	93	92	474	40.7/-/35.8
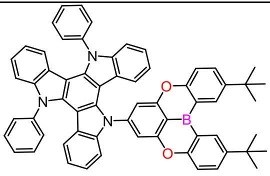 TDBA-DI	4	99	89	-	38.15/-/34.03
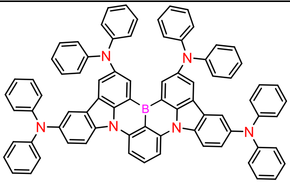 HF BN3	5	-	99	558	40.5/-/32.4

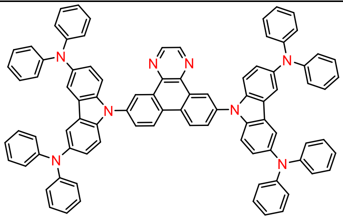 DQBC	6	95	92	534	39.1/36.1/29.1
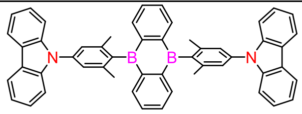 CzDBA	7	100	84	528	38.0/-/37.9

3. Supplemental Methods

3.1 General Information. 5-phenyl-5,11-dihydroindolo[3,2-b]carbazole and 2-(4-bromophenyl)-4,6-diphenyl-1,3,5-triazine were purchased from the Suzhou ge'ao New Material Co Ltd. All other reagents used for reaction, purification and measurements were purchased from Shanghai Bide Medical Technology Co. Ltd. All other reagents used for device fabrication were purchased from Jilin Optical and Electronic Materials Co. Ltd. These materials were received and used without further purification. The other reagents and solvents were used as received from commercial sources without further purification. 600 MHz ^{1}H-NMR and 150 MHz ^{13}C-NMR spectra were measured by a JEOL JNM-ECS600 spectrometer at room temperature in deuterated dichloromethane or chloroform respectively with tetramethyl silane as the internal standard. ^{11}B, ^{19}F solid-state NMR spectrum was obtained on a JNM-ECZ600R spectrometer, with a tube diameter of 3.2 mm, a frequency of 12 kHz. MALDI-TOF-MS data was performed on a Shimadzu AXIMA Performance MALDI-TOF instrument in positive detection modes.

3.2 Computational methods. The calculations were performed with the Gaussian 09 package,[8] using the density functional theory (DFT) and time-dependent density functional theory (TD-DFT) method.[9-11] The ground-state structures were fully optimized by the B3LYP functional with the 6 31G(d,p) basis set. The S_n and T_n energies and spin-orbit couplings (SOC S_n-T_n) were calculated using a TD-DFT approach at the B3LYP/6-31G(d,p) level. The natural transition orbital (NTO) analysis of S_1 and T_1 states was carried out using the Multiwfn software.[12] The SOC matrix elements are computed using the ORCA package[13]. The optical simulation of OLED device was performed by commercial software OFSS 1.0 (Wuhan Yuwei Optical Software Co., Ltd.). The input parameters include refractive index value, extinction

coefficient, the thickness of each layer values (all measured by ellipsometry), as well as photoluminescence spectrum, PLQY, and the ratio of horizontal emitting dipole orientation ($\Theta_{//}$) of the emitting layer.

3.3 Measurement of absorption and emission characteristics. 1×10^{-5} M solutions were prepared by stepwise dilution for solution measurements. Thin films for photophysical characterization were prepared by thermal evaporation on quartz substrates at 1-2 Å sec^{-1} in a vacuum chamber with a base pressure of < 10^{-5} torr. UV-vis absorption and PL spectra were measured using UV-2600 (Shimadzu) and FluoroMax-4P (Horiba) instruments at 77 and 298 K. The PLQYs were obtained with an absolute photoluminescence quantum yield measurement system Hamamatsu C9920-03G in an integrating sphere. The solution sample was bubbled with nitrogen for 10 minutes before measurement while the films were measured in air. The transient spectra were collected on an Edinburgh Fluorescence Spectroscopy FLS1000.

3.4 Ultraviolet photoelectron spectroscopy characterizations. The ultraviolet photoelectron spectroscopy (UPS) characterizations were carried out on AXIS ULTRA DLD with monohromatized HeI radiation at 21.2 eV. The sample films were prepared by vacuum deposition upon ITO substrates with sheet resistances of about 15 **Ω**, the thickness of the film was 10 nm, and measured at a bias voltage of -9 V inside a vacuum chamber at 3.0×10^{-8} Torr.

3.5 The rate constants calculation. The prompt fluorescence and delayed fluorescence quantum yield ratio (Φ_{PF} and Φ_{DF}) were determined from the total PL quantum efficiency (Φ_{PL}) and the proportion of the integrated area of each component in the transient spectra to the total integrated area, r_{PF} and r_{DF} are individual component ratio for prompt and delayed fluorescence. The quantum efficiencies and rate constants were determined using the following equations according to Adachi's method.[14]

(1) $\Phi_{PF} = \Phi_{PL} r_{PF}$ $\quad r_{PF} = \tau_1 A_1/(\tau_1 A_1 + \tau_2 A_2)$

(2) $\Phi_{DF} = \Phi_{PL} r_{DF}$ $\quad r_{DF} = \tau_2 A_2/(\tau_1 A_1 + \tau_2 A_2)$

(3) $k_r = \Phi_{PF}/\tau_{PF}$

(4) $k_{ISC} = (1 - \Phi_{PF})/\tau_{PF}$

(5) $k_{RISC} = \Phi_{DF}/(k_{ISC} \cdot \tau_{PF} \cdot \tau_{DF} \cdot \Phi_{PF})$

3.6 Determination of the emitting dipole orientation. To determine emitting dipole orientation of an emitting film, angle-resolved and polarization-resolved PL measurements

were performed by Hamamatsu's established molecular orientation measurement system (C14234-11, Hamamatsu Photonics). The sample consisted of a fused silica substrate with a 10-nm-thick film doped with emitters. The sample was attached to a fused silica half-cylinder prism by index matching liquid. The excitation of the samples was performed with the 360-nm line of the continuous-wave laser with a fixed excitation angle of 45°. The angle-dependent p-polarized emission intensity at the peak wavelength of the PL spectrum of the emitting layer was detected. The emitting dipole orientation was then determined by least-square fitting of the measured angle-dependent polarized emission intensity with calculated results.

3.7 Device fabrication and measurement of EL characteristics. All compounds were subjected to temperature-gradient sublimation under a high vacuum before use. OLEDs were fabricated on the ITO-coated glass substrates with multiple organic layers sandwiched between the transparent bottom indium-tin-oxide (ITO) anode and the top metal cathode. Before device fabrication, the ITO glass substrates were pre-cleaned carefully. All material layers were deposited by vacuum evaporation in a vacuum chamber with a base pressure of 10^{-6} torr. The deposition system permits the fabrication of the complete device structure in a single vacuum pump-down without breaking the vacuum. The deposition rate of organic layers was kept at 0.1~0.2 nm s^{-1}. The doping was conducted by co-evaporation from separate evaporation sources with different evaporation rates. The current density, voltage, luminance, external quantum efficiency, electroluminescent spectra and other characteristics were measured with a Keithley 2400 source meter and an absolute EQE measurement system in an integrating sphere at the same time. The EQE measurement system is Hamamatsu C9920-12, which is equipped with Hamamatsu PMA-12 Photonic multichannel analyzer C10027-02 whose longest detection wavelength is 1100 nm. The Lambertian distribution was measured under a constant voltage of 4.5 V using a light distribution measurement system (Hamamatsu C9920-11). The angle range was set from 0° to +90° with a step of 5°.

3.8 Synthesis

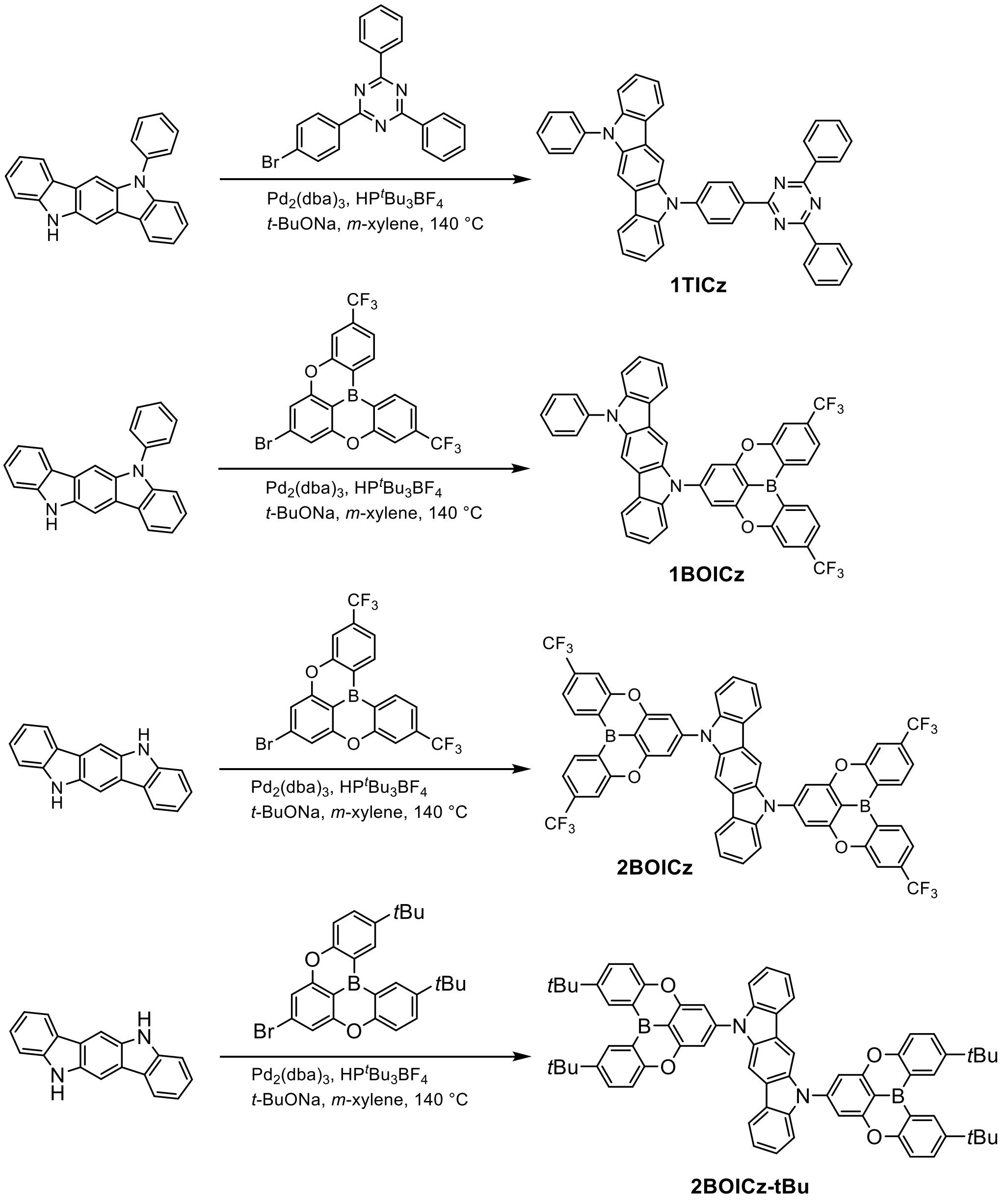

Synthesis of 7-bromo-3,11-bis(trifluoromethyl)-5,9-dioxa-13b-boranaphtho[3,2,1-de]anthracene (**BO-Br**): This compound was synthesized according to the previous work[7] and obtained as solid, yield 30%. ^{1}H NMR (600 MHz, Chloroform-*d*) δ (ppm): 8.66 (s, 2H), 7.77 (s, 2H), 7.61 (s, 2H), 7.40 (s, 2H).

Synthesis of 5-(4-(4,6-diphenyl-1,3,5-triazin-2-yl)phenyl)-11-phenyl-5,11-dihydroindolo[3,2-b]carbazole (**1TICz**): Compound **1TICz** was synthesized according to the previous work[8] and obtained as solid, yield 60%. ^{1}H NMR (600 MHz, Methylene Chloride-d_2) δ (ppm): 9.09 (d, *J* = 8.1 Hz, 2H), 8.84 (d, *J* = 7.3 Hz, 4H), 8.27 (s, 1H), 8.13 (t, *J* = 6.8 Hz, 2H), 8.10 (s, 1H), 7.97 (d, *J* = 8.1 Hz, 2H), 7.71 – 7.58 (m, 11H), 7.56 – 7.51 (m, 1H), 7.45 – 7.38 (m, 3H), 7.28 – 7.22 (m, 2H). ^{13}C NMR (151 MHz, Methylene Chloride-d_2) δ (ppm): 176.62, 175.64, 171.86, 171.11, 157.31, 149.18, 148.99, 142.17, 141.60, 136.51, 136.27,

134.69, 132.73, 130.76, 130.09, 128.98, 128.78, 127.41, 127.26, 126.80, 126.30, 126.20, 124.14, 123.98, 123.76, 123.43, 120.33, 120.25, 120.00, 119.52, 109.77, 109.56, 100.20, 100.02. MALDI-TOF: Calculated: 639.2423, Found: 639.1927. Anal. Calcd (%) for $C_{45}H_{29}N_5$: C, 84.48; H, 4.57; N, 10.95; Found: C, 84.20; H, 4.67; N, 11.13.

Synthesis of 5-(3,11-bis(trifluoromethyl)-5,9-dioxa-13b-boranaphtho[3,2,1-de]anthracen-7-yl)-11-phenyl-5,11-dihydroindolo[3,2-b]carbazole (**1BOICz**): A mixture of 5-phenyl-5,11-dihydroindolo[3,2-b]carbazole (1.0 g, 3.00 mmol), 7-bromo-3,11-bis(trifluoromethyl)-5,9-dioxa-13b-boranaphtho[3,2,1-de]anthracene (1.60 g, 3.29 mmol), $Pd_2(dba)_3$ (200 mg, 0.22 mmol), [(*t*-Bu)$_3$PH][BF_4] (208 mg, 0.72 mmol) and *t*-BuONa (0.69 g, 7.18 mmol) in *m*-xylene (60 mL) was stirred at 140 °C under N_2 atmosphere for 24 h. After cooling to room temperature, the mixture was washed with brine and dried with anhydrous sodium sulfate. After removing the solvent, the mixture was purified by column chromatography on silica gel using petroleum ether/dichloromethane (5:1, v/v) as an eluent to give the pure product as a yellow powder, 1.55 g, yield 70%. The compound was further purified by temperature-gradient sublimation under vacuum (train sublimation). ^{1}H NMR (600 MHz, Methylene Chloride-d_2) δ (ppm): 8.71 (d, J = 8.0 Hz, 2H), 8.28 (s, 1H), 8.08 (d, J = 7.8 Hz, 1H), 7.99 (d, J = 7.7 Hz, 1H), 7.93 (s, 1H), 7.73 (s, 2H), 7.71 – 7.64 (m, 7H), 7.62 (d, J = 8.0 Hz, 2H), 7.54 (t, J = 7.3 Hz, 1H), 7.40 (t, J = 7.6 Hz, 1H), 7.35 (d, J = 3.6 Hz, 2H), 7.24 (t, J = 7.4 Hz, 1H), 7.20 (dt, J = 7.9, 4.0 Hz, 1H). ^{13}C NMR (151 MHz, Methylene Chloride-d_2) δ (ppm): 160.29, 158.56, 145.64, 142.17, 141.21, 138.17, 137.57, 136.01, 135.43, 135.36, 135.15, 130.07, 127.43, 127.18, 126.42, 126.30, 124.73, 124.57, 124.37, 124.04, 123.52, 123.34, 122.76, 120.62, 120.36, 120.15, 119.59, 119.30, 115.93, 109.81, 109.59, 100.26, 100.11. MALDI-TOF: Calculated: 736.1757, Found: 736.1260. Anal. Calcd (%) for $C_{44}H_{23}BF_6N_2O_2$: C, 71.76; H, 3.15; B, 1.47, F, 15.48, N, 3.80, O, 4.34; Found: C, 71.58; H, 3.23; B, 1.26, F, 15.69, N, 3.73, O, 4.51.

Synthesis of 5,11-bis(3,11-bis(trifluoromethyl)-5,9-dioxa-13b-boranaphtho[3,2,1-de]anthracen-7-yl)-5,11-dihydroindolo[3,2-b]carbazole (**2BOICz**): Compound **2BOICz** was synthesized according to the same procedure described above for the synthesis of **1BOICz**. After removing the solvent in the vacuum, the solid materials were washed with hexane, and collected by filtration. The obtained solid was sonicated with methanol, collected by filtration to give the title compound **2BOICz** a blight yellow solid. This compound was further purified by temperature-gradient sublimation under vacuum to afford highly pure materials, yield 50%. ^{1}H NMR, ^{13}C NMR could not be measured because of low solubility. ^{1}H NMR, ^{13}C NMR could not be measured because of low solubility. ^{11}B solid-state NMR (192 MHz) δ (ppm): 22.55,

33.40. ^{19}F solid-state NMR (564 MHz) δ (ppm): −50.01. MALDI-TOF: Calculated: 1064.1887, Found: 1064.2141. Anal. Calcd (%) for $C_{58}H_{26}B_2F_{12}N_2O_4$: C, 65.45; H, 2.46; B, 2.03, F, 21.42, N, 2.63, O, 6.01; Found: C, 65.21; H, 2.56; B, 2.33, F, 21.70, N, 2.41, O, 5.89.

Synthesis of 5,11-bis(2,12-di-tert-butyl-5,9-dioxa-13b-boranaphtho[3,2,1-de]anthracen-7-yl)-5,11-dihydroindolo[3,2-b]carbazole (**2BOICz-tBu**): Compound **2BOICz-tBu** was synthesized according to the same procedure described above for the synthesis of **2BOICz**. After removing the solvent in the vacuum, the solid materials were washed with hexane, and collected by filtration. The obtained solid was sonicated with methanol, collected by filtration to give the title compound **2BOICz-tBu**, yield 40%. ^{1}H NMR (600 MHz, Methylene Chloride-*d_2*) δ (ppm): ^{1}H NMR (400 MHz, Methylene Chloride-*d_2*) δ 8.84 (d, J = 2.5 Hz, 4H), 8.40 (s, 2H), 8.17 (d, J = 7.9 Hz, 2H), 7.84 (dd, J = 8.8, 2.4 Hz, 4H), 7.70 (d, J = 8.2 Hz, 2H), 7.61 (s, 4H), 7.55 (d, J = 8.7 Hz, 4H), 7.44 (t, J = 7.7 Hz, 2H), 7.27 (t, J = 7.5 Hz), 1.51(s, 36H). MALDI-TOF: Calculated: 1016.4896, Found: 1016.4651. Anal. Calcd (%) for $C_{70}H_{62}B_2N_2O_4$: C, 82.68; H, 6.15; B, 2.13; N, 2.75; O, 6.29; Found: C, 82.57; H, 6.21; B, 2.06; N, 2.70; O, 6.35.

4. Supplementary References

1. Wang, Q., Xu, Y., Yang, T., Xue, J. & Wang, Y. Precise Functionalization of a Multiple-Resonance Framework: Constructing Narrowband Organic Electroluminescent Materials with External Quantum Efficiency over 40%. *Adv. Mater.* **35**, e2205166 (2023).

2. Jiang, P. et al. Quenching-Resistant Multiresonance TADF Emitter Realizes 40% External Quantum Efficiency in Narrowband Electroluminescence at High Doping Level. *Adv. Mater.*, e2106954 (2021).

3. Naveen, K. R. et al. Achieving High Efficiency and Pure Blue Color in Hyperfluorescence Organic Light Emitting Diodes using Organo-Boron Based Emitters. *Adv. Funct. Mater.* **32**, 2110356 (2021).

4. Ahn, D. H. et al. Highly efficient blue thermally activated delayed fluorescence emitters based on symmetrical and rigid oxygen-bridged boron acceptors. *Nat. Photonics* **13**, 540-546 (2019).

5. Hu, Y. X. et al. Efficient selenium-integrated TADF OLEDs with reduced roll-off. *Nat. Photonics* **16**, 803-810 (2022).

6. Chen, Y. et al. Approaching Nearly 40% External Quantum Efficiency in Organic Light Emitting Diodes Utilizing a Green Thermally Activated Delayed Fluorescence Emitter with an Extended Linear Donor-Acceptor-Donor Structure. *Adv. Mater.* **33**, e2103293 (2021).

7. Wu, T.-L. et al. Diboron compound-based organic light-emitting diodes with high efficiency and reduced efficiency roll-off. *Nat. Photonics* **12**, 235-240 (2018).

8. M. J. Frisch, G. W. Trucks, H. B. Schlegel, G. E. Scuseria, M. A. Robb, et al. Gaussian 16, Revision B.01, Gaussian, Inc., Wallingford CT, 2016.

9. J.-D. Chai and M. H.-Gordon. Long-range corrected hybrid density functionals with damped atom–atom dispersion corrections. *Phys. Chem. Chem. Phys.* **10**, 6615-6620 (2008).

10. L. Kronik, T. Stein, S. Refaely-Abramson, R. Baer. Excitation Gaps of Finite-Sized Systems from Optimally Tuned Range-Separated Hybrid Functionals. *J. Chem. Theory Comput.* **8**, 1515-1531 (2012).

11. H. Sun, C. Zhong, J. L. Brédas. Reliable Prediction with Tuned Range-Separated Functionals of the Singlet–Triplet Gap in Organic Emitters for Thermally Activated Delayed Fluorescence. *J. Chem. Theory. Comput.* **11**, 3851-3858(2015).

12. T. Lu, F. Chen. Multiwfn: A multifunctional wavefunction analyzer. *J. Comput. Chem.* **33**, 580-592 (2012).

13. F. Neese. The ORCA program system. *WIREs Comput. Mol. Sci.* **2**, 73-78 (2012).

14. Masui, K., Nakanotani, H. & Adachi, C. Analysis of exciton annihilation in high-efficiency sky-blue organic light-emitting diodes with thermally activated delayed fluorescence. *Organic Electronics* **14**, 2721-2726 (2013).
